# High-resolution genetic map and SNP chip for molecular breeding in *Panax ginseng,* a tetraploid medicinal plant

**DOI:** 10.1093/hr/uhae257

**Published:** 2024-09-09

**Authors:** Woohyeon Cho, Woojong Jang, Hyeonah Shim, Jiseok Kim, Youngju Oh, Jee Young Park, Young Chang Kim, Jung-Woo Lee, Ick-Hyun Jo, Misun Lee, Jinsu Gil, Martin Mascher, Murukarthick Jayakodi, Xuejiao Liao, Jiang Xu, Deqiang Dou, Yi Lee, Tae-Jin Yang

**Affiliations:** Department of Agriculture, Forestry and Bioresources, Plant Genomics and Breeding Institute, College of Agriculture & Life Sciences, Seoul National University 1 Gwanak-ro, Gwanak-gu, Seoul 08826, Republic of Korea; Herbal Medicine Resources Research Center, Korea Institute of Oriental Medicine, 111, Geonjae-ro, Naju, Jeollanam-do 58245, Republic of Korea; Department of Agriculture, Forestry and Bioresources, Plant Genomics and Breeding Institute, College of Agriculture & Life Sciences, Seoul National University 1 Gwanak-ro, Gwanak-gu, Seoul 08826, Republic of Korea; Department of Agriculture, Forestry and Bioresources, Plant Genomics and Breeding Institute, College of Agriculture & Life Sciences, Seoul National University 1 Gwanak-ro, Gwanak-gu, Seoul 08826, Republic of Korea; Department of Agriculture, Forestry and Bioresources, Plant Genomics and Breeding Institute, College of Agriculture & Life Sciences, Seoul National University 1 Gwanak-ro, Gwanak-gu, Seoul 08826, Republic of Korea; Department of Agriculture, Forestry and Bioresources, Plant Genomics and Breeding Institute, College of Agriculture & Life Sciences, Seoul National University 1 Gwanak-ro, Gwanak-gu, Seoul 08826, Republic of Korea; Department of Herbal Crop Research, National Institution of Horticultural and Herbal Science, Rural Development Administration, Eumseong 27709, Republic of Korea; Department of Herbal Crop Research, National Institution of Horticultural and Herbal Science, Rural Development Administration, Eumseong 27709, Republic of Korea; Department of Crop Science and Biotechnology, Dankook University, Cheonan 31116, South Korea; Department of Industrial Plant Science and Technology, Chungbuk National University, Cheongju 28644, Republic of Korea; Department of Industrial Plant Science and Technology, Chungbuk National University, Cheongju 28644, Republic of Korea; Leibniz Institute of Plant Genetics and Crop Plant Research (IPK), Gatersleben, Seeland 06466, Germany; Department of Soil and Crop Sciences, Texas A&M AgriLife Research-Dallas, Dallas, Texas, USA; Institute of Chinese Materia Medica, China Academy of Chinese Medical Sciences, Beijing 100700, China; Institute of Chinese Materia Medica, China Academy of Chinese Medical Sciences, Beijing 100700, China; College of Pharmacy, Liaoning University of Traditional Chinese Medicine, Dalian 116600, China; Department of Industrial Plant Science and Technology, Chungbuk National University, Cheongju 28644, Republic of Korea; Department of Agriculture, Forestry and Bioresources, Plant Genomics and Breeding Institute, College of Agriculture & Life Sciences, Seoul National University 1 Gwanak-ro, Gwanak-gu, Seoul 08826, Republic of Korea

## Abstract

Ginseng (*Panax ginseng*) renowned as the king of medicinal plants. Ginseng grows slowly under shade conditions, requiring at least 4 years to produce a limited number of seeds. Molecular breeding of ginseng faces challenges due to its the tetraploid genome and the absence of an efficient molecular marker system. To overcome these obstacles, we adopted genotyping-by-sequencing to delve into genetic mapping and survey genetic diversity. We constructed a comprehensive genetic map comprising 24 linkage groups, each corresponding to one of the 24 chromosomes in the ginseng genome, based on 1216 nonredundant SNPs obtained from an F_**2**_ mapping population. Additionally, 431 103 SNPs were identified from 119 diverse ginseng genotypes. From these, 192 informative subgenome-specific single copy SNPs were selected to develop a SNP chip. The SNP chip was used to genotype a large ginseng collection, encompassing registered cultivars, breeding lines, wild-simulated ginseng, and wild ginseng from various countries and regions. We evaluated the utility of the assay for molecular breeding with 919 ginseng genotypes. This breeder-friendly SNP chip promises versatility, enabling purity assessments of seeds and products, the authentication of species and cultivars, and the determination of homozygosity and homogeneity rates for breeding lines. Genotype data for 1200 ginseng genotypes are now stored in our database. This SNP chip lays the foundation for a molecular breeding in ginseng and will facilitate the breeding process in this medicinal crop.

## Introduction

*Panax ginseng* C. A. Meyer has garnered global recognition for its pharmacological efficacy and economic significance [1]. In the current era, where consumer health is a prevailing trend, ginseng has gained substantial public interest [2]. However, the inherent biological attributes of ginseng pose significant challenges. The plant demands a minimum of 4 years to reach the next generation and produces a limited number of offspring during each reproductive cycle [3]. Additionally, ginseng is highly susceptible to environmental influences, making it a challenge to maintain consistent growth conditions for individual plants. These intricate characteristics render traditional hybrid breeding in ginseng a laborious and time-intensive process. Moreover, ginseng is prone to causing continuous cropping obstacles, which often necessitates the change of planting locations between generations. This further complicates the traditional trait determination. Consequently, the imperative need for a molecular breeding system has become increasingly evident, to expedite the breeding timeline and nurture the development of high-quality ginseng cultivars.

Recent studies have shown compelling evidence indicating that the ginseng genome has undergone two whole genome duplication events [4]. The most recent genome duplication, a relatively recent occurrence, has given rise to the complex allotetraploid genome structure of ginseng, characterized by a chromosome count of 2*n* = 4*x* = 48 [5] and an estimated genome size exceeding 3.6 Gbp [4]. It has been established that a significant portion of the ginseng genome (80%) is occupied by repetitive elements [6]. Notably, the presence of paralogous regions, a consequence of the recent duplication event, has posed considerable challenges for researchers delving into the intricacies of the complex ginseng genome [7]. Future studies must employ methods that can accurately overcome these challenges to pave the way for the development of a robust molecular breeding system.

With the rapid advancement of sequencing technologies and related research tools, various innovative methods for genomic research have emerged [8]. The genotyping-by-sequencing (GBS) method stands out as an effective tool for the analysis of large genomes cost-effectively. GBS leverages the use of restriction enzymes to construct a reduced representation library, effectively mitigating the complexity inherent to large genomes [9]. This reduction in the number of sequenced regions serves to increase read coverage, thereby enhancing the accuracy of genotype calling data and reducing the proportion of missing data [10]. Furthermore, the GBS protocol, which incorporates the use of two different restriction enzymes, offers a more efficient means of focusing on restricted sequences, facilitating the analysis of large genomes [11]. GBS analysis enables the discovery of sequence variants, in a high-throughput manner, particularly of single nucleotide polymorphisms (SNPs), and has the potential to yield large-scale molecular DNA markers for crop breeding applications [12].

A genetic map is a crucial tool in genetic and breeding research, especially for identifying qualitative and quantitative trait loci that play a vital role in crop improvement. Although several chromosome-level genome sequences are reported for ginseng [13, 14], genetic map composed of credible SNP markers are unavailable up to now. DNA markers provide valuable insights for various genomic analyses, including studies on diversity [15], genetic mapping [15], cultivar authentication [14], and marker-assisted selection (MAS) [16]. The combination of a genetic map and DNA marker information can generate a synergistic effect in crop improvement. Moreover, there is a pressing need for a reference genetic map in ginseng that can guide the chromosome-scale scaffolding of genome sequence assemblies. Such a map would be invaluable for advancing our understanding of ginseng genetics and facilitating the development of improved ginseng varieties.

Various molecular markers have been developed for *P. ginseng,* including techniques like random amplified polymorphic DNA (RAPD), simple sequence repeat (SSR), derived cleaved amplified polymorphic sequences (dCAPS), and insertion and deletion (InDel) markers [17]. However, many of these markers were designed without considering allotetraploid nature of the ginseng genome, leading to reduced reliability because they confound paralogous sequences [18]. Furthermore, these markers are based on labor-intensive and time-consuming gel electrophoresis. In recent times, new technologies such as Kompetitive Allele-Specific PCR (KASP), TaqMan, Fluidigm SNP chip, and Affymetrix Axiom have been developed for high-throughput genotyping [19]. These technologies offer the advantage of cost, time, and labor efficiency by enabling the simultaneous analysis of numerous samples. These technologies have found applications in various crops, including rice, pumpkin, melon, cucumber, and wheat, aimed at improving crop breeding and enhancing agricultural practices.

**Table 1:** Summary of GBS sequencing

Purpose	Material	Restriction enzyme	Sequencing			
			Platform	Read type	Total reads	Total base pairs
Genetic map	YP, CP, 100 F_2_ individuals	EcoRI + MseI	Illumina HiSeq 2000	Single end	684 996 546	69 184 651 146
		NsiI + MseI	Illumina HiSeq 2000	Paired end	1 511 785 644	152 690 350 044
		HindIII + MseI	Illumina HiSeq X	Single end	578 575 070	87 364 835 570
Genome diversity	119 Ginseng collections	PstI + MseI	Illumina HiSeq 2000	Single end	281 225 762	27 278 898 914
		EcoRI + MseI	Illumina HiSeq 2000	Single end	643 290 559	61 755 893 664

In this study, we tried to develop a useful molecular breeding tool, well guided by genome sequences and genetic map. A high-resolution genetic map was constructed for the first time in ginseng from the thousands of SNPs produced by GBS analysis. Subsequently, we successfully developed a high-throughput genotyping system that leverages KASP, TaqMan and Fluidigm technologies to detect fluorescence signals. This system was applied to diverse ginseng genetic resources, including cultivars, breeding lines, wild ginseng and wild-simulated ginseng which refers to ginseng plants that are intentionally grown under conditions resembling the wild natural environment. These SNP chips represent a vital and practical tool for advancing digital breeding techniques aimed at enhancing ginseng breeding and development of ginseng industry.

## Results

### Genetic map construction

The mapping population was generated by crossing the ginseng cultivars Chunpoong (CP) and Yunpoong (YP). CP and YP are the first registered varieties in Korea. Among them, we published a genome assembly paper on CP in 2018 [4]. These two cultivars are still being cultivated and show distinct agricultural traits. CP has poor growth but excellent processing characteristics, while YP is resistant to light, forms multiple stems, and has excellent growth and high yield [20]. Due to their genetic stability and significant differences in agricultural traits, the Rural Development Administration (RDA, Eumseong, Korea) developed an F_2_ population between these cultivars, which we utilized in our study. Given that ginseng requires at least 10 years to establish an F_2_ population due to its long generation time, we used this pre-established population.

Three GBS libraries were constructed in order to generate SNP data for a F_2_ population between YP and CP (Table 1). Following demultiplexing based on barcode sequences, a total of 1622 million (M) trimmed reads were utilized for mapping to the reference genome. A total of 35.7% of the reads that mapped to multiple locations were discarded. The number of reads uniquely mapped to each sample ranged from 1.4 M to 24.2 M, with an average of 10.2 M. Finally, 1039 M reads were utilized for the calling of high-throughput SNP data. Initially, 615 812 raw variants were identified. To ensure the selection of a suitable SNPs for genetic mapping, several filtering steps were implemented. A total of 10 440 SNPs were finally used in the construction of a high-density genetic map.

**Figure 1:**
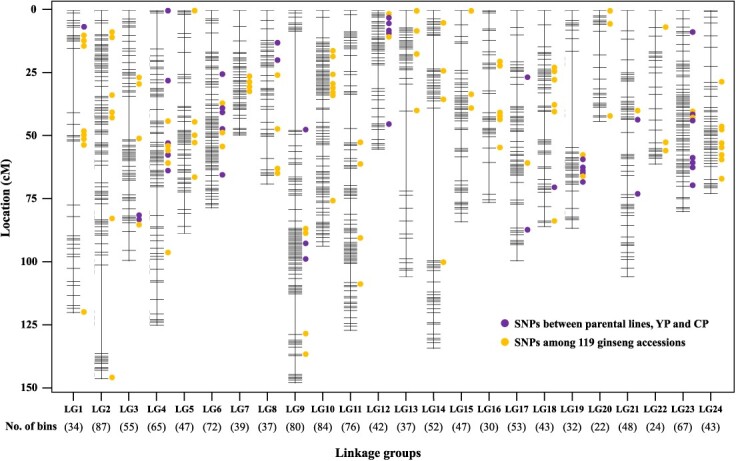
Genetic linkage map composed of 1216 non-redundant markers and distribution of SNP chip markers. The 24 linkage groups have been arranged based on an unpublished chromosome-level genome assembled in our laboratory. The linkage group numbers are ordered from the pseudo-chromosome sequences, largest to the smallest, for sub-genome A (LG1–12) and sub-genome B (LG13–24). The dots indicate the relative positions of 186 single-copy SNP chip (Table 3). The 90 dots indicate SNPs identified between YP and CP mapping population and the other 96 dots indicate SNPs identified from GBS data of 119 ginseng collections.

Following the initial ordering of 10 440 SNPs, any incorrectly assigned genotypes were thoroughly reviewed and manually corrected. With the updated genotype data, SNP binning was carried out in order to create a nonredundant dataset. As a result, a total of 1216 nonredundant SNPs were selected to construct a genetic map (Table S1). The 24 linkage groups, each representing one of the 24 chromosomes, were successfully obtained (Fig. 1). The total map length was 2196.54 cM with the smallest linkage group of LG20, which containing 22 markers spanning a length of 43.34 cM, and the largest linkage group of LG9, which containing 80 markers spanning a length of 145.31 cM (Table 2). The mean distance between adjacent markers in each linkage group exhibited a range from 1.07 cM on LG6 to 3.47 cM on LG1, with an average of 1.92 cM.

**Table 2:** Information of genetic map

Linkage group	No. of bins^*^	No. of SNPs	Length (cM)	Mean distance (cM)
1	34	61	117.91	3.47
2	87	716	143.72	1.65
3	55	125	97.56	1.77
4	65	409	122.82	1.89
5	47	362	87.10	1.85
6	72	1208	77.02	1.07
7	39	627	48.68	1.25
8	37	84	67.73	1.83
9	80	778	145.31	1.82
10	84	1514	91.96	1.10
11	76	258	124.83	1.64
12	42	232	54.17	1.29
13	37	115	104.09	2.81
14	52	352	131.84	2.54
15	47	278	82.50	1.76
16	30	72	75.00	2.50
17	53	268	97.69	1.84
18	43	195	84.40	1.96
19	32	394	85.05	2.66
20	22	102	43.34	1.97
21	48	214	103.94	2.16
22	24	116	59.93	2.50
23	67	994	78.50	1.17
24	43	891	71.45	1.66
Total	1216	10 365	2196.54	1.92

a Bin: number of non-redundant SNPs.

As telomere-to-telomere genome sequence is available [13], we have examined the overlap between the linkage groups and the genome. Out of the 1216 SNPs used to construct the genetic map, 1211 were found to exactly match to the 24 chromosome sequences of the genome. The remaining 5 SNPs appear to be located in unplaced contigs. Each linkage group corresponds to a specific chromosome, indicating both a high quality of the genome assembly and the accuracy of the genetic map (Fig. S1).

### SNP chip development

**Figure 2:**
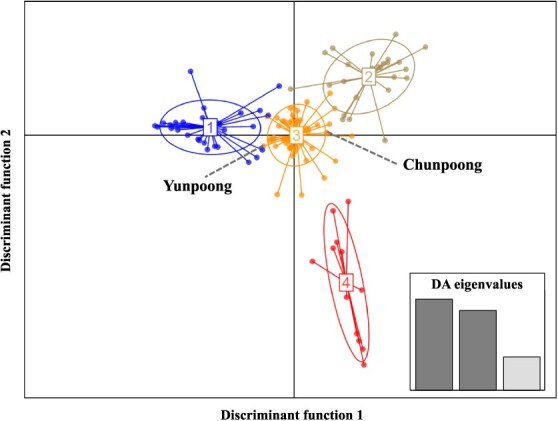
DAPC scatterplot analysis of 119 ginseng accessions based on 5000 SNPs randomly selected from the GBS data. The graph illustrates individuals as dots and represents groups using inertia ellipses. During the analysis, three discriminant eigenvalues were selected to elucidate the relationships among the clusters. The axes represent the first two linear discriminants. Each number depicted corresponds to a distinct subpopulation as identified through DAPC analysis.

A total of 925 M (89 Gbp) reads were generated from 119 ginseng individuals by sequencing two GBS libraries (Table 1). Following the filtering process, 7165 informative SNPs were obtained (Fig. S2). Among them, 5000 SNPs randomly selected for discriminant analysis of principal components (DAPCs) to assess the diversity of the genetic pool (Fig. 2). A total of 119 individuals dispersed widely and K-means clustering identified four distinct groups.

Out of 7165 informative SNPs, 323 single-locus unique SNPs were identified, which detect sub-genome unique targets. Among single-locus unique SNPs, 14 of them are randomly selected and developed into KASP markers for validation (Table S2, S3). These markers were then applied to the same 119 ginseng genotypes that had been used for GBS sequencing (Table S4). Each KASP marker displayed three distinct genotype clusters, which indicating two homozygous genotypes and one heterozygous genotype, on the endpoint fluorescence scatter plot (Fig. S3). Ultimately, a final set of 192 assays was selected for SNP chip, exhibiting precise and consistent signals accompanied by dense endpoint clusters (Table S5, S6).

The final 192 SNPs comprise 186 nuclear genome-derived SNPs and six plastid genome-derived SNPs (Table 3). Among the 186 nuclear genome-derived SNPs, 90 were found to be identical to the SNP markers included in the genetic map (Fig. 1, purple dots). Furthermore, the approximate positions of 96 SNPs that were polymorphic in the 119 ginseng accessions were estimated and marked on the genetic map (Fig. 1, orange dots). The wide distribution of the 192 SNPs across the genetic map provides compelling evidence of the genome-wide screening capabilities afforded by the SNP chip. Also, 186 SNPs derived from the nuclear genome were mapped across the 24 chromosomes of the telomere-to-telomere genome sequence [13] (Table S7). The assays were divided into two sets, each containing 96 assays, based on genetic distance calculated using the genotyping results to minimize genetic information bias between sets (Fig. S4).

**Table 3:** Genome position and statistics of 192 SNP markers on the chip assays

Genome	SNP regions			Major allele frequency	Gene diversity	Heterozygosity	PIC value
	Total	Genic^a^	Non-genic				
Nuclear	186	123	63	0.5041–0.9931	0.0138–0.5000	0–0.6928	0.0137–0.3750
Plastid	6	6	-	0.8043–0.9931	0.0137–0.3148	0–0.0035	0.0137–0.2653
Mean				0.7787	0.3035	0.0903	0.2464

a SNPs derived from exon, intron, and 5 kb flanked region

The SNP chip was applied to a diverse set of 919 ginseng genotypes (Tables S4 and S8). The genotype results were used to calculate the statistics for each marker (Table 3). The major allele frequency ranged from 0.5041 to 0.9931, with an average of 0.7787, while gene diversity ranged from 0.0137 to 0.5000, with an average of 0.3035. Observed heterozygosity varied from 0 to 0.6928, with an average of 0.0903, and polymorphic information content (PIC) values ranged from 0.0137 to 0.3750, with an average of 0.2464.

### Genetic diversity and homozygosity of ginseng germplasms

**Figure 3:**
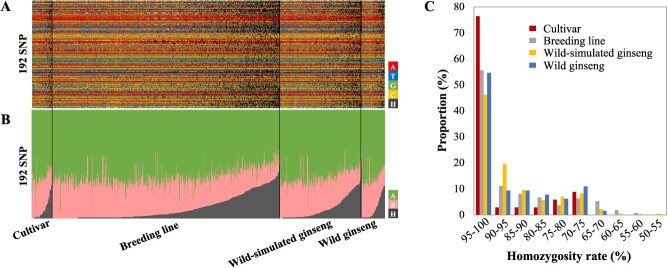
Genotype information for 192 SNP loci across 919 ginseng germplasms. (A) Genotypes of 919 collections (X-axis) for 192 SNP locus. The A, T, G, C, and heterozygous genotypes for 192 SNP loci (Up panel, Y axis). (B) The proportion of homozygous alleles in each individual. The major and minor type homozygous genotypes and heterozygous alleles were depicted (low panel, Y axis). (C) Homozygosity rate for individuals belonged different populations.

The heterozygosity rate was calculated for 192 SNP loci across the collections of cultivars, breeding lines, wild-simulated ginseng and wild ginseng (Table S9, Fig. 3). In the majority of the genotypes, the genotype of the reference genome (green color) predominated (Fig. 3A). Commercial cultivars exhibited higher homozygosity rates than those of other genotypes (Fig. 3B). The majority of individuals within the categories of cultivars, breeding lines, wild-simulated ginseng, and wild ginseng displayed high homozygosity rates, rendering them suitable candidates for the rapid development of inbred cultivars. Specifically, 76.5% of cultivars, 55.6% of breeding lines, 46.3% of wild-simulated ginseng, and 54.7% of wild ginseng exhibited homozygosity levels above 95%. Additionally, 15.1% of cultivars, 11.3% of breeding lines, 3.5% of wild-simulated ginseng, and 1.6% of wild ginseng showed 100% homozygosity. Conversely, 23.5% of cultivars, 36.1% of breeding lines, 50.2% of wild-simulated ginseng, and 43.8% of wild ginseng displayed heterozygosity levels ranging from 5% to 30%.

### Population structure analysis

**Figure 4:**
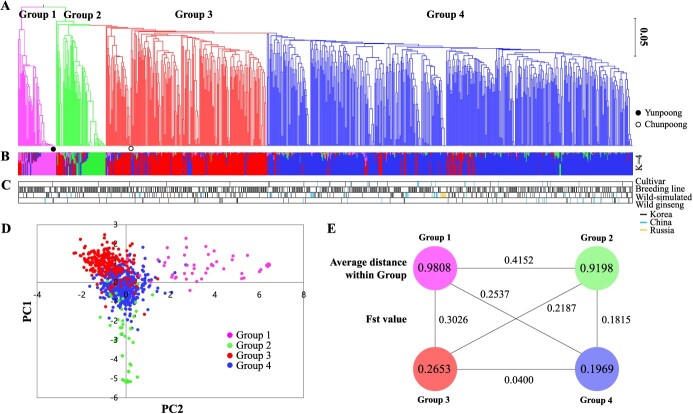
Population genetic analysis of 919 ginseng germplasms. (A) UPGMA phylogenetic tree, (B) population structure plot, (C) position information for each population, and (D) PCA analysis plot. Collections from Korea, China, and Russia are indicated. (E) Mean Fst value of each group and genetic distance between groups.

A phylogenetic tree was constructed and genetic relationships were examined through principal component analysis (PCA) in the panel of 919 genotypes (Fig. 4A, 4D). Population structure analysis was also performed, and the ΔK method supported the presence of four genetically distinct clusters (i.e., K = 4; Fig. 4B, S5), referred to as Groups 1–4 (Table S10). This is consistent with the findings of the phylogenetic tree and PCA. Individuals from each category of cultivar, breeding line, wild-simulated ginseng, and wild ginseng were all intermingled across the groups with no discernible pattern (Fig. 4C). The distribution of wild ginseng from Korea and China across various groups suggests the absence of genetic barriers between the two countries. In contrast, those from Russia were found to predominantly cluster in Group 4, which may be indicative of a shared origin from a single collection site.

The origin of the plants did not influence the grouping, which can be attributed to several factors. First, it could be because the genetic differences observed may not strictly correlate with the geographical origins but rather reflect a blended genetic background due to factors such as historical trade. Second, the 919 ginseng samples are primarily cultivated plants, not wild resources, and these cultivated plants can be traded between countries as seed, further blending their genetic backgrounds. Third, we could not exclude the possibility of the SNP bias from the 192 SNPs. We also have primary phenotypic data for 119 genotypes (Table S11); however, no significant factors were identified that corresponded to the observed grouping patterns (Table S12, Fig. S6). The grouping pattern might be explained by underlying genetic structure, which could be revealed through more detailed genomic analysis or the inclusion of additional phenotypic traits not yet measured.

With regard to the genetic distance of groups, Groups 1 and 2 exhibited a high average distance within group of 0.9808 and 0.9198, respectively (Fig. 4E). In contrast, Groups 3 and 4 exhibited low average distances within the groups, at 0.2653 and 0.1969, respectively. The mean value of the fixation index (Fst) for Group 1 and 2 was 0.4152, indicating a significant differentiation between populations and a lack of shared genetic diversity against the other groups (Fig. 4E). Group 3 and 4 exhibited the smallest Fst value of 0.0400, indicating the presence of numerous shared genotypes. The genetic distance between the groups is consistent with the results of phylogenetic and PCA analysis.

### Case study for molecular breeding; identification of homozygosity and homogeneity for the breeding population

The SNP chip was employed to genotype breeding accessions, which are maintained by pedigree selection. Two individuals were examined for each of 250 accessions, comprising six accessions of *Panax quinquefolius* and 244 accessions of *P. ginseng*, to understand the homozygosity and homogeneity of the accessions. The homozygosity of 500 individuals was identified by counting homozygous genotype calls among 192 SNP sites. The homogeneity of 250 accessions was determined by counting identical genotype calls between two individuals of each accession.

**Figure 5:**
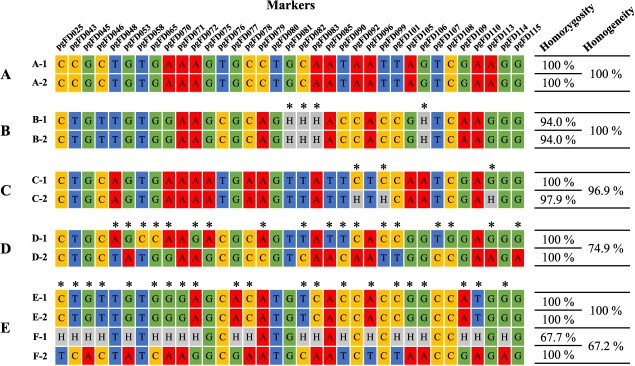
Case study for application of SNP chip across inbreeding population which bred and maintained by pureline selection. Five accessions (A–E) were inspected and two individuals from each accession were randomly selected and genotyped by SNP chip. Homozygous genotypes were represented as A, T, G, or C and heterozygous genotypes were represented as H. Individuals showed different genotypes were denoted as *. Homozygosity was revealed for each individual and homogeneity was revealed for each accession by comparing genotypes of two individuals.

A number of different scenarios were encountered in relation to the homozygosity and homogeneity (Fig. 5). Firstly, seventeen accessions displayed complete homozygosity and homogeneity. For example, accession A showed 100.0% homozygous and uniform genotypes across 192 SNP sites, indicating a genetically stable and potentially cultivable state. Secondly, two accessions were observed having over 95.0% for both homozygosity and homogeneity. In accession B, for example, one individual exhibited 100.0% homozygosity, while the other exhibited 97.9%. In this case, proper selection and seed harvest from the individuals exhibiting high homozygosity will promote the development of an inbred line. Thirdly, three accessions were found having high homozygosity, over 99.0%, yet displaying a homogeneity below 80.0%. For instance, in accession C, both genotypes exhibited 100.0% homozygosity, but showed a notably low level of homogeneity, at just 74.9%. It can be postulated that seeds from two homozygous lines were inadvertently mixed maintained as a single accession. Lastly, a cross-pollinated individual among the breeding lines could be selected based on its heterozygosity. One individual of accession E (E-1) exhibited a low level of homozygosity of 67.7%, whereas another individual (E-2) exhibited 100.0% homozygosity (E-2). It can be postulated that the E-1 individual was derived by cross-pollination during the previous generation. On the basis of genotype, it can be inferred that accession D is the most probable pollen donor of the E-1 individual. This is because the two accessions share the same homozygous alleles at the 33.3% locus, while the other heterozygous alleles can be attributed to the combination of D and E.

### SNP chip development and accuracy assessment between genotyping methods

In this study, we employed a genotyping approach that involved the use of GBS, a SNP chip, and KASP markers. The resulting genotype data were then subjected to a comparative analysis (Table S13). The genotypes obtained from the SNP chip and KASP marker were identical, indicating a high level of consistency. However, among the 2737 genotyping results, 55 instances of GBS data exhibited discrepancies from the other methods. Genotyping errors in GBS analysis are a recognized challenge, and it’s noteworthy that other GBS studies have also reported lower heterozygosity values than the actual ones [21]. The discrepancies were predominantly categorized into two groups: the first involved heterozygous genotypes in assays (SNP chip and KASP) but homozygous genotypes in GBS, while the second entailed homozygous genotypes in assays but heterozygous genotypes in GBS. The first case, observed 39 times, is likely attributable to sequencing bias at sites with heterozygous alleles. The second case, observed 16 times, is likely attributable to sequencing or mapping errors of some GBS reads. Both errors are further compounded by low read coverage of GBS data for certain duplicated targets.

## Discussion

### Utility of the first genetic map

The construction of a genetic map for ginseng presents significant challenges due to its complex genetic nature. Firstly, ginseng has a tetraploid genome complicating the segregation and inheritance patterns [22]. Secondly, individual ginseng plant produces approximately 40 seeds after 4 years of growth, leading to low seed production rates [23]. Thirdly, it is difficult to maintain plant viability over extended periods [24, 25].

Despite the availability of genome sequences, the absence of a comprehensive genetic map significantly hinders the broad application of the genome data. Without the foundational framework of a genetic map, interpreting and applying genomic data becomes significantly constrained. A high-quality genetic map can assist in orientating and coordinating ginseng genome sequences, especially for the correcting of structural errors introduced during the Hi-C assembly. Furthermore, the development of a genetic map is of great importance for expanding pan-genome research, which aims to understand the complete set of genes within a species [26]. In recent years, pan-genome studies have unveiled significant amounts of structural variations within species [27]. Genetic map will enhance the resolution and accuracy of pan-genome analyses by providing the detailed genetic contexts.

The genetic map is essential for marker development and molecular breeding. The genetic map guide development of molecular markers related to agronomic traits. Quantitative trait loci (QTLs) linked to desirable agronomic traits or medicinal effects, such as yield, root size, and secondary metabolites, are influenced by multiple genes and environmental factors, making them complex for breeding. The map can provide frame for genetic mapping of QTLs using mapping population and also for genome wide association study using large collections. Markers associated to agronomic characteristics will improves marker-assisted breeding, enhancing the molecular breeding to develop elite ginseng cultivars.

### The null genotype may reflect the indel events from the pan-genomes

The allotetraploid *P. ginseng* has undergone recent whole genome duplication events, resulting in the presence of highly similar paralogous sequences [4]. While various molecular markers have been developed for ginseng [17], the process of genotyping has often been confounded by highly similar paralogous sequences, which has led to the formation of multiple amplicons. A well-known example of polyploidy in plants is bread wheat, which possesses an allohexaploid genome where repetitive and paralogous sequences occupy over 70% of the genome [28]. Similar to wheat, the SNP markers developed in this study underwent a rigorous selection process involving diverse filtering steps to exclude SNPs detected from paralogous regions, ensuring the development of single locus targeting sub-genome specific markers [29]. In selecting the SNPs, we opted for clarity in genotyping by exclusively selecting biallelic SNPs, those with only two types of alleles at the designated site. Molecular markers are typically designed to amplify sequences containing either the reference or alternative allele.

Allelic drop-out, often referred to as allelic drop, is a phenomenon that can occur during the PCR amplification process, particularly in the context of genotyping [30]. This issue arises by various factors, such as primer-site mutations, suboptimal PCR conditions, and low DNA quantity or quality. However, it could also be interpreted as indicative of the presence of a third allele. As pangenome studies have arisen, the importance of genomic research utilizing diverse gene pools is being increasingly emphasized [31]. With regard to the molecular marker application, there are numerous opportunities to encounter germplasms with unexpected genotypes. Hence, the genotyped result labelled as “Null” should not be regarded as an error, but rather as a potential indication of a third allele. Rather than dismissing unamplified alleles, they should be considered carriers of homozygous indel variation.

### Cross-species SNP chip application

The divergence between *P. ginseng* and *P. quinquefolius* occurred less than one million years ago, and the two species exhibit similar morphological characteristics (Fig. S7D) [32]. We applied SNP chip for *P. quinquefolius* and *P. vietnamensis* individuals. The individuals of each species formed a distinct cluster that was separate from the *P. ginseng* groups (Figs S7A, S7B). A significant disparity was observed in the number of null genotypes between *P. ginseng*, *P. quinquefolius* and *P. vietnamensis*. *P. ginseng* individuals have a mean value of 3.31 and a median value of 2 (0–5), *P. quinquefolius* has a mean value of 36.94 and a median value of 38 (31–41), and *P. vietnamensis* has a mean value of 115 and a median value of 116 (109–120) (Fig. S7C). In our previous study, phylogenetic analysis based on the chloroplast genomes of the *Panax* species provided insights into the divergence times of each species (Fig. S7D) [4].

The number of null genotypes observed from each species corresponds to their respective divergence times. Furthermore, given that ginseng is an allotetraploid [33], it can be categorized into subgenome A and subgenome B. The subgenome B is more closely related to the diploid *Panax* species [14]. The incidence of null genotypes of *P. vietnamensis* was compared across the two subgenomes of *P. ginseng*. Out of the 192 SNPs, 110 were located in subgenome A and 72 SNPs were located in subgenome B. In subgenome A, an average of 77 SNPs (approximately 70%) exhibited null genotypes, while an average of 35 SNPs (approximately 50%) showed null genotypes in subgenome B. This observation may be attributed to the closer similarity of the *P. vietnamensis* genome to subgenome B. These findings not only suggest the potential utility of an SNP chip in identifying the species roughly, but also underscore a novel approach to interpreting the null genotypes.

### SNP chip database and molecular breeding

In our previous study, we constructed the Ginseng Genome Database (http://ginsengdb.snu.ac.kr/) [34]. Subsequently, the database has been expanded to include a SNP chip database, accessible via the same web address. To date, we have applied to more than 1200 ginseng individuals collected from cultivars, landraces, and mountain ginseng in Korea, China, and Russia. All genotyping results obtained via the SNP chip application will be stored and made accessible, along with providing clustering analysis services. This facilitates the assessment of genetic diversity and the establishment of a classification system.

Moreover, the SNP chip can assist in maintaining the purity of cultivars and can be utilized to protect breeder’s rights by providing a cultivar-specific marker system. We could develop a cultivar-specific marker sets with a few SNP markers which can differentiate from all other ginseng collections. For instance, we developed a marker set by selecting a few reliable markers from 192 SNPs to identify cultivar “Geumsun” and “Cheonryang” from others (Table S14).

Ginseng is generally considered as a predominantly self-pollinating plant, with approximately 4% of hybrids occurring in its natural environment [35]. Since ginseng bears seeds from its fourth year, reducing the generation number is the most essential step to obtain a homozygous inbred line [3]. Traditionally, ginseng breeding has been predominantly conducted through the pure line selection method. This involves the selection of superior individuals from the local landrace population and further self-fertilization over several generations [36]. Most of registered cultivars has high homozygosity, but 23.5% exhibited heterozygosity levels ranging from 5% to 30%, indicating potential challenges in maintaining purity. Ensuring the purity of registered cultivars is crucial to preserve their superior characteristics. The utilization of the SNP chip will greatly facilitate this process in an efficient manner.

Furthermore, the SNP chip can be utilized to monitor the genotypes of the entire genome and verify the genetic homogenization in breeding lines. Selection of individuals with higher homozygosity will significantly reduce the breeding period. Selection of breeding lines with 100% homozygosity and 100% homogeneity will verify the genetic fixation as an inbred line, which can be evaluated as a candidate for cultivar registration. Eventually, this invaluable and informative SNP chip and its database represent a significant advancement in the establishment of a molecular breeding system for ginseng.

## Materials and methods

### Plant materials and DNA extraction

Genetic mapping was conducted using a F_2_ population. The mapping population was generated by crossing the ginseng cultivars Chunpoong (CP) and Yunpoong (YP). The mapping population consists of 100 F_2_ individuals. In addition to the mapping population, diverse ginseng germplasm, a total of 119 collections comprising 14 cultivars and 105 breeding lines, provided by RDA were also utilized for GBS analysis to identify a large scale SNPs for genetic diversity analysis.

The developed SNP markers were applied to 919 ginseng germplasms, including 18 cultivars and 593 ginseng breeding lines from RDA, 171 wild-simulated ginseng germplasms provided by Chungbuk National University, 73 germplasms collected from various regions in Korea, Russia, and China, and 64 germplasms provided by China Academy of Chinese Medical Sciences (Table S4, S8). Fresh leaves were frozen in liquid nitrogen and then ground using a mortar and pestle. Genomic DNA was isolated using a modified cetyltrimethylammonium bromide (CTAB) method [37]. The quantity and quality of the extracted DNA were measured using a NanoDrop ND-1000 (Thermo scientific Inc., Wilmington, DE, USA). For polymerase chain reaction (PCR) analysis and the construction of GBS libraries, the DNA concentration of each sample was adjusted to 10 ng/ul and 80 ng/ul, respectively.

### GBS library construction and sequencing

In the construction of GBS libraries, double digestion was performed to effectively reduce the genome complexity of the large ginseng genome. For genetic map construction, three combination of restriction enzymes, *EcoRI/MseI*, *NsiI/MseI*, and *HindIII/MseI* were used. An *EcoRI/MseI* GBS library was prepared for single-end sequencing, using the parents and a population of 100 F_2_ individuals. On the other hand, *NsiI/MseI* and *HindIII/MseI* libraries were prepared for paired-end sequencing, using parents and 94 F_2_ individuals.

For SNP chip development, *PstI*/*MseI* and *EcoRI*/*MseI* restriction enzyme combinations were used. Single-end sequencing was conducted for both libraries using the Illumina HiSeq 2000 platform. The GBS libraries were constructed according to the previously published protocol [11] and provided to Macrogen (Seoul, Korea) for sequencing.

### SNP calling and filtering

A draft sequence of 3.0 Gbp with 9788 scaffolds with an N50 of 570 Kbp was completed and we used this sequence as reference [4, 34]. Reference genome was indexed using BWA [38], SAMTools [39], and Picard (http://broadinstitute.github.io/-picard/) program. Raw sequence data was demultiplexed based on individual specific barcode sequence using sabre program (https://github.com/najoshi/sabre) and trimmed with the criteria of quality score ≥ 20 and read length ≥ 80 using Trimmomatic ver. 0.33 [40]. Trimmed data was mapped to ginseng reference genome using BWA-MEM program [41], and uniquely mapped reads were selected and sorted by SAMTools program. After read grouping with Picard program, SNP calling and filtering were conducted using GATK Unified Genotyper v3.4–46 [42] and VCFtools program [43] (Fig. S2).

For genetic map construction, 92 F_2_ individuals were used, excluding eight individuals with extensive missing data due to limited sequenced reads. Missing data were addressed through the use of imputation programs suitable for non-model organisms lacking genomic resources. InDel and low-quality SNPs (<Q30) were removed, and biallelic SNPs with a minor allele frequency of ≥0.05 were selected from the raw variants. SNPs that had another SNP within 100 bp were excluded, and only those having a homozygous genotype in both parents with a minimum read depth of 3 were selected. For each progeny, SNPs with missing data of ≤70% and mapped reads depth of ≥5 were finally selected for genetic mapping using the VCFtools program. Additionally, 8 out of 100 F2 individuals with missing data of >70% were excluded.

For SNP chip development, we eliminating low-quality reads and barcode sequences and obtained 729 M reads, which constitute approximately 78.9% of the total reads. The high-quality reads were mapped to reference genome, resulting in 430 M (58.9%) reads being mapped to unique site, 297 M (40.8%) reads being mapped to multiple sites, and 2 M (0.3%) reads remaining unmapped. Only reads that mapped to unique sites were utilized for variant calling. InDel and low-quality SNPs (<Q30) were removed, and only biallelic SNPs were selected. For each individual, SNPs with a minor allele frequency of ≥0.1, missing data of ≤90% and mapped reads depth of ≥3 were finally selected. Additionally, SNPs with another SNP within 50 bp were excluded. A total of 431 103 raw variants were identified, among which 249 885 were SNPs. By filtering SNPs with minimum reads depth of 3 and 90% threshold for missing data, we obtained 81 855 SNPs. Following a series of filtering processes, 7165 informative SNPs were obtained (Fig. S2).

### Discriminant analysis of principal components with GBS data

Discriminant analysis of principal components (DAPC) was conducted to assess the genetic diversity of 119 accessions. Randomly selected 5000 informative SNPs were used. DAPC implemented with the “adegenet” package in R and visualized through scatterplots [44].

### PCR amplification

With the candidate SNPs for the SNP chip, flanking 150 bp sequence of SNPs are extracted and aligned to ginseng reference genome using BLASTN program [45]. With the SNPs that flanking sequence is matched to single locus of reference genome, assay was designed using PrimerBlast program [46]. High resolution melting analysis (HRM) was used to validate single-target amplification through tm calling analysis. PCR amplification was conducted in 20 μl reactions containing 1 unit of Taq polymerase (Inclone Biotech, Seongnam, Korea), 2 μl of 10x reaction buffer, 0.4 μl of 10 mM dNTPs, 20 ng genomic DNA, 10 pmole of each primer, and 1 μl of 0.05 mM syto9 green fluorescent nucleic acid (Invitrogen, Waltham, MA, USA). The thermal cycling and plate reading were performed using a Roche LC480 (Roche Diagnostics, Penzberg, Germany). The thermal cycling conditions were as follows: 95°C for 5 min, 45 cycles of 95°C for 20 s, 59°C for 20 s, and 72°C for 20 s, 1 cycle of 95°C for 1 min, 1 cycle of 40°C for 1 min, 60°C for 5 s, and 95°C (ramp rate, 0.02°C/s), and 1 cycle of 40°C for 30 s.

### KASP marker development and genotyping

In contrast to multi-plex marker such as SNP chip, KASP is a single-plex marker with the advantage of being able to apply a single marker on a small scale. KASP marker was developed for validation of SNPs. PCR amplification was conducted in 10 μl volume of reactions containing 5 μl of 2× KASP Master Mix (LGC Genomics, Hoddesdon, UK), 0.14 μl of Assay mix, 20 ng genomic DNA, and 2.86 μl of nuclease-free water. The thermal cycling and plate reading were performed using a Roche LC480 (Roche Diagnostics, Penzberg, Germany). The thermal cycling conditions were as follows: 94°C for 15 min, 10 cycles of 94°C for 20 s, 61–55°C (touchdown 0.6°C per cycle) for 60 s, 26 cycles of 94°C for 20 s, and 55°C for 60 s. For more denser cluster, additional cycle was performed as recommended by LGC genomics with the conditions of: 3 cycles of 94°C for 20 s and 57°C for 60 s, and 1 cycle of 30°C for 60 s.

### Fluidigm SNP chip development and high-throughput genotyping

Position information and flanking sequence of validated SNPs were uploaded to D3 assay design website (http://d3.fluidigm.com/) to design optimum assay. Fluidigm genotyping was performed following SNP Genotyping protocol (Fluidigm, PN 68000098 Q1). Genotyping was performed with 94 ginseng germplasms for assay validation using 96.96 Dynamic Array IFCs (Fluidigm, CA, South San Francisco, USA). For experiment, IFC controller, FC1 Cycler, and EP1 Reader was used (Fluidigm, CA, South San Francisco, USA). The genotyping results were revised manually with Fluidigm SNP genotyping analysis program. Assays that precisely cluster samples and having at least two genotypes are final selected.

### Genetic diversity analysis with SNP chip genotyping result

Major allele frequency, gene diversity, heterozygosity, and polymorphism information content (PIC) value, which implying the informativity of molecular marker were calculated using PowerMarker v3.25 program [47]. The phylogenetic and population structure analysis was performed with 919 ginseng germplasms based on genotype results of 192 SNP sites. Also, to take a closer look of genetic diversity of ginseng existing in Northeast Asia, population analysis were conducted with wild ginseng individuals collected from Russia, China, and diverse region of Korea. The phylogenetic analysis was conducted based on the unweighted pair group method with arithmetic mean (UPGMA) method using PowerMarker v3.25 program. With genetic distance statistics, dendrogram was drawn using MEGA v6.0 program [48]. The population structure was presumed using STRUCTURE software [49] for subpopulation number estimation and the values of K were set from two to seven. Simulations were run with 100 000 burn-in period, 100 000 Markov Chain Monte Carlo (MCMC) repeats, and five independent iterations. The optimal number of subpopulations was determined by the highest peak in the Delta K graph. Bar plots of STURCTURE analysis were rendered using the STRUCTURE Plot V2.0 program [50]. 919 individuals were sorted according to clustering analysis.

For cross-species SNP application result, phylogenetic analysis and population structure analysis were conducted with the same method with the analysis conducted only with ginseng individuals.

## Supplementary Material

Web_Material_uhae257

## Data Availability

All the data generated or analyzed in this study are included in this published article and its supplementary information files. All the sequence data of the present study have been deposited in the NCBI Sequence Read Archive (SRA) database under BioProject PRJNA1062125 and PRJNA1026738.
