# Supplementary material for: High-resolution genetic map and SNP chip for molecular breeding in Panax ginseng, a tetraploid medicinal plant
Source: Hortic Res. 2024 Sep 9;11(12):uhae257. doi: 10.1093/hr/uhae257 (PMC11630301; doi:10.1093/hr/uhae257)

**Table S2.** Summary of KASP markers developed and used in this study.

| **No.** | **KASP ID** | **Fluidigm ID** | **Locus** | **Position** | **Region type** | **Related gene** | **Development** |
| --- | --- | --- | --- | --- | --- | --- | --- |
| 1 | pgKASP001 | PgFD131 | Pg_scaffold2156 | 250,052 | Exon | Pg_S2156.7 | Jang 2019 |
| 2 | pgKASP003 | PgFD132 | Pg_scaffold0429 | 625,474 | 3' 1kb | Pg_S0429.5 | Jang 2019 |
| 3 | pgKASP004 | PgFD133 | Pg_scaffold1005 | 229,194 | 3' 1kb | Pg_S1005.5 | Jang 2019 |
| 4 | pgKASP005 | PgFD134 | Pg_scaffold1335 | 196,636 | Intron | Pg_S1335.18 | Jang 2019 |
| 5 | pgKASP006 | PgFD135 | Pg_scaffold5398 | 77,489 | Intron | Pg_S5398.9 | Jang 2019 |
| 6 | pgKASP007 | PgFD136 | Pg_scaffold1400 | 260,817 | Non-genic |  | Jang 2019 |
| 7 | pgKASP008 | PgFD137 | Pg_scaffold2132 | 449,182 | 5' 3kb | Pg_S2132.9 | Jang 2019 |
| 8 | pgKASP010 | PgFD139 | Pg_scaffold3506 | 168,603 | Non-genic |  | Jang 2019 |
| 9 | pgKASP011 | PgFD140 | Pg_scaffold5500 | 94,483 | 3' 3kb | Pg_S5500.2 | Jang 2019 |
| 10 | pgKASP013 | PgFD001 | Pg_scaffold0008 | 15,761 | Non-genic |  | In this study |
| 11 | pgKASP014 | PgFD003 | Pg_scaffold0027 | 86,915 | Non-genic |  | In this study |
| 12 | pgKASP015 | PgFD005 | Pg_scaffold0341 | 410,098 | Non-genic |  | In this study |
| 13 | pgKASP016 | PgFD006 | Pg_scaffold1176 | 590,369 | Non-genic |  | In this study |
| 14 | pgKASP017 | PgFD007 | Pg_scaffold1308 | 289,384 | Non-genic |  | In this study |
| 15 | pgKASP018 | PgFD008 | Pg_scaffold1344 | 225,508 | Non-genic |  | In this study |
| 16 | pgKASP019 | PgFD009 | Pg_scaffold1399 | 497,893 | Non-genic |  | In this study |
| 17 | pgKASP020 | PgFD010 | Pg_scaffold1459 | 100,158 | Non-genic |  | In this study |
| 18 | pgKASP021 | PgFD011 | Pg_scaffold1496 | 203,469 | Non-genic |  | In this study |
| 19 | pgKASP022 | PgFD012 | Pg_scaffold1523 | 553,046 | Non-genic |  | In this study |
| 20 | pgKASP023 | PgFD016 | Pg_scaffold2569 | 355,719 | Intron | Pg_S2569.27 | In this study |
| 21 | pgKASP026 | PgFD020 | Pg_scaffold4490 | 6,558 | 3' 4kb | Pg_S4490.1 | In this study |
| 22 | pgKASP028 | PgFD022 | Pg_scaffold5203 | 93,436 | Non-genic |  | In this study |
| 23 | pgKASP029 | PgFD024 | Pg_scaffold5603 | 47,086 | Intron | Pg_S5603.2 | In this study |

**Table S3.** Information of KASP markers developed in this study.

| **Marker** | **FAM** | **HEX** | **Allele X/Y Primer Seq** | | **Common Primer Seq** |
| --- | --- | --- | --- | --- | --- |
| pgKASP013 | T | C | FAM | CCGAGCACTCCCTCCTCTTCT | CAAGGAGGGGTTGGTTCGACAATAT |
|  |  |  | HEX | CGAGCACTCCCTCCTCTTCC |  |
| pgKASP014 | A | C | FAM | GCAGCAGAATTCAAAGAATGCAAA | CTTCCTTTGTAATCCGGGCGTAGAT |
|  |  |  | HEX | CTGCAGCAGAATTCAAAGAATGCAAC |  |
| pgKASP015 | T | C | FAM | GACAAAGTTAACCCAGTGTCTAGCA | AACCTGCCAGGATGACCCAGATTTT |
|  |  |  | HEX | ACAAAGTTAACCCAGTGTCTAGCG |  |
| pgKASP016 | A | T | FAM | TCCCCCTCCAATTCTGCCAAT | CGTCTAACAAGAAGAGTGGGATGCAT |
|  |  |  | HEX | CTTCCCCCTCCAATTCTGCCAAA |  |
| pgKASP017 | A | G | FAM | ATGACCTTTCTATAGGAGCTACGTAA | CTATAAGACTCAGCAGTCATCTATCACAA |
|  |  |  | HEX | GACCTTTCTATAGGAGCTACGTAG |  |
| pgKASP018 | G | A | FAM | CCTCACACTGTCTGTAAAGCAAC | CTAGGCTGCAAGATTGCTTGCTTGAT |
|  |  |  | HEX | AGCCTCACACTGTCTGTAAAGCAAT |  |
| pgKASP019 | A | T | FAM | GCCAGGGCACTATGCTCATTCA | CCCGACGCTAGGATATAGGGATTT |
|  |  |  | HEX | GCCAGGGCACTATGCTCATTCT |  |
| pgKASP020 | T | C | FAM | AACGTCCACAAGATTCACTACAAGAAA | AAGGAATATTCTGTCGGAATTAGGCCTT |
|  |  |  | HEX | CGTCCACAAGATTCACTACAAGAAG |  |
| pgKASP021 | A | C | FAM | CGACGGATACCTCTCTGTACTTTT | CCTTGGTTGGATTTACTGGGGTGAA |
|  |  |  | HEX | GACGGATACCTCTCTGTACTTTG |  |
| pgKASP022 | G | A | FAM | GGAACCGAACTAGGAGACGC | CCAGAATTCCTCCTCCACCAAGAAA |
|  |  |  | HEX | GGGAACCGAACTAGGAGACGT |  |
| pgKASP023 | A | G | FAM | TCATCTATGCATTTCTCAGTCTCGTT | GTATGAGTTCAACACCAATTGGGCGAA |
|  |  |  | HEX | CATCTATGCATTTCTCAGTCTCGTC |  |
| pgKASP026 | A | T | FAM | GCACTGCGGCGCCAGGCA | GCGTCAGAATCATTACACAAAACTGCAAA |
|  |  |  | HEX | GCACTGCGGCGCCAGGCT |  |
| pgKASP028 | A | G | FAM | AGGATGAGGAGGCAACAAGTTTTTTT | CACATACTCAATCTGCTGCAGTTCATATA |
|  |  |  | HEX | GGATGAGGAGGCAACAAGTTTTTTC |  |
| pgKASP029 | T | C | FAM | GCAGATATATAATGGATTTCATCACGAATA | CATCGCACATTGCTATAAAATCCTACCAT |
|  |  |  | HEX | CAGATATATAATGGATTTCATCACGAATG |  |

**Table S5.** Position information and statistics of 192 SNP chip assays.

| **Genome** | **Marker** | **Locus** | **Position** | **Region type** | **Related gene** | **MAF** ^a^ | **GD** ^b^ | **He** ^c^ | **PIC** ^d^ |
| --- | --- | --- | --- | --- | --- | --- | --- | --- | --- |
| Nuclear | PgFD001 | Pg_scaffold0008 | 15761 | Non-genic |  | 0.5572 | 0.4934 | 0.1181 | 0.3717 |
|  | PgFD002 | Pg_scaffold0023 | 615426 | Non-genic |  | 0.8445 | 0.2626 | 0.0636 | 0.2281 |
|  | PgFD003 | Pg_scaffold0027 | 86915 | Non-genic |  | 0.5359 | 0.4974 | 0.1620 | 0.3737 |
|  | PgFD005 | Pg_scaffold0341 | 410098 | Non-genic |  | 0.5182 | 0.4993 | 0.1466 | 0.3747 |
|  | PgFD006 | Pg_scaffold1176 | 590369 | Non-genic |  | 0.6706 | 0.4418 | 0.1297 | 0.3442 |
|  | PgFD007 | Pg_scaffold1308 | 289384 | Non-genic |  | 0.6847 | 0.4318 | 0.1886 | 0.3386 |
|  | PgFD008 | Pg_scaffold1344 | 225508 | Non-genic |  | 0.6595 | 0.4491 | 0.1232 | 0.3483 |
|  | PgFD009 | Pg_scaffold1399 | 497893 | Non-genic |  | 0.8013 | 0.3184 | 0.0961 | 0.2677 |
|  | PgFD010 | Pg_scaffold1459 | 100158 | Non-genic |  | 0.5357 | 0.4975 | 0.1556 | 0.3737 |
|  | PgFD011 | Pg_scaffold1496 | 203469 | Non-genic |  | 0.5744 | 0.4889 | 0.1477 | 0.3694 |
|  | PgFD012 | Pg_scaffold1523 | 553046 | Non-genic |  | 0.6098 | 0.4759 | 0.1607 | 0.3626 |
|  | PgFD014 | Pg_scaffold1718 | 44145 | Exon | ID=Pg_S1718.1 | 0.5227 | 0.4990 | 0.0014 | 0.3745 |
|  | PgFD016 | Pg_scaffold2569 | 355719 | Intron | ID=Pg_S2569.27 | 0.8620 | 0.2379 | 0.0658 | 0.2096 |
|  | PgFD017 | Pg_scaffold3108 | 25029 | Non-genic |  | 0.6363 | 0.4629 | 0.6928 | 0.3557 |
|  | PgFD018 | Pg_scaffold3443 | 178819 | Non-genic |  | 0.7830 | 0.3398 | 0.0961 | 0.2821 |
|  | PgFD020 | Pg_scaffold4490 | 6558 | 3' 4kb | ID=Pg_S4490.1 | 0.6549 | 0.4520 | 0.1065 | 0.3499 |
|  | PgFD022 | Pg_scaffold5203 | 93436 | Non-genic |  | 0.8111 | 0.3064 | 0.1139 | 0.2595 |
|  | PgFD024 | Pg_scaffold5603 | 47086 | Intron | ID=Pg_S5603.2 | 0.6723 | 0.4406 | 0.1320 | 0.3435 |
|  | PgFD025 | Pg_scaffold6567 | 92505 | Non-genic |  | 0.5041 | 0.5000 | 0.2044 | 0.3750 |
|  | PgFD026 | Pg_scaffold0015 | 916640 | 5' 5kb | ID=Pg_S0015.25 | 0.8292 | 0.2832 | 0.0725 | 0.2431 |
|  | PgFD027 | Pg_scaffold0098 | 499961 | Non-genic |  | 0.8859 | 0.2022 | 0.1147 | 0.1818 |
|  | PgFD028 | Pg_scaffold0285 | 195941 | Non-genic |  | 0.9312 | 0.1281 | 0.0312 | 0.1199 |
|  | PgFD029 | Pg_scaffold0344 | 39146 | Non-genic |  | 0.8825 | 0.2075 | 0.1298 | 0.1859 |
|  | PgFD030 | Pg_scaffold0381 | 706887 | Non-genic |  | 0.8874 | 0.1998 | 0.0589 | 0.1799 |
|  | PgFD031 | Pg_scaffold0632 | 505878 | Non-genic |  | 0.9162 | 0.1536 | 0.0497 | 0.1418 |
|  | PgFD032 | Pg_scaffold0800 | 733579 | 3' 1kb | ID=Pg_S0800.5 | 0.8239 | 0.2902 | 0.0866 | 0.2481 |
|  | PgFD033 | Pg_scaffold0809 | 316401 | Non-genic |  | 0.9352 | 0.1212 | 0.0417 | 0.1139 |
|  | PgFD034 | Pg_scaffold1334 | 5672 | 3' 4kb | ID=Pg_S1334.1 | 0.8490 | 0.2565 | 0.0729 | 0.2236 |
|  | PgFD035 | Pg_scaffold1726 | 484198 | Non-genic |  | 0.9268 | 0.1358 | 0.0288 | 0.1265 |
|  | PgFD038 | Pg_scaffold2196 | 344398 | 5' 1kb | ID=Pg_S2196.13 | 0.7714 | 0.3527 | 0.1247 | 0.2905 |
|  | PgFD039 | Pg_scaffold2242 | 70448 | Non-genic |  | 0.7656 | 0.3589 | 0.0000 | 0.2945 |
|  | PgFD040 | Pg_scaffold3746 | 224991 | Non-genic |  | 0.8751 | 0.2185 | 0.0509 | 0.1947 |
|  | PgFD043 | Pg_scaffold6165 | 10120 | Non-genic |  | 0.8770 | 0.2157 | 0.0585 | 0.1924 |
|  | PgFD045 | Pg_scaffold6494 | 10125 | NA |  | 0.9242 | 0.1401 | 0.0453 | 0.1303 |
|  | PgFD046 | Pg_scaffold0050 | 1869085 | Non-genic |  | 0.8055 | 0.3134 | 0.0964 | 0.2643 |
|  | PgFD047 | Pg_scaffold0057 | 1213063 | Non-genic |  | 0.8653 | 0.2332 | 0.0557 | 0.2060 |
|  | PgFD048 | Pg_scaffold0124 | 1292187 | Non-genic |  | 0.6524 | 0.4535 | 0.1409 | 0.3507 |
|  | PgFD049 | Pg_scaffold0199 | 867144 | Non-genic |  | 0.9221 | 0.1436 | 0.0334 | 0.1333 |
|  | PgFD050 | Pg_scaffold0239 | 514225 | Non-genic |  | 0.6822 | 0.4336 | 0.1142 | 0.3396 |
|  | PgFD051 | Pg_scaffold0248 | 148184 | Non-genic |  | 0.7166 | 0.4062 | 0.1185 | 0.3237 |
|  | PgFD052 | Pg_scaffold0251 | 778445 | 5' 1kb | ID=Pg_S0251.4 | 0.6338 | 0.4642 | 0.1142 | 0.3565 |
|  | PgFD053 | Pg_scaffold0277 | 755966 | Non-genic |  | 0.7407 | 0.3841 | 0.1181 | 0.3103 |
|  | PgFD054 | Pg_scaffold0321 | 545351 | 5' 1kb | ID=Pg_S0321.10 | 0.5948 | 0.4820 | 0.1491 | 0.3659 |
|  | PgFD055 | Pg_scaffold0396 | 395036 | 3' 2kb | ID=Pg_S0396.5 | 0.6935 | 0.4251 | 0.1147 | 0.3347 |
|  | PgFD056 | Pg_scaffold0403 | 763412 | 3' 1kb | ID=Pg_S0403.39 | 0.5162 | 0.4995 | 0.1319 | 0.3747 |
|  | PgFD057 | Pg_scaffold0430 | 854842 | 5' 4kb | ID=Pg_S0430.3 | 0.6988 | 0.4209 | 0.1121 | 0.3323 |
|  | PgFD058 | Pg_scaffold0490 | 426407 | Non-genic |  | 0.8080 | 0.3103 | 0.0937 | 0.2622 |
|  | PgFD059 | Pg_scaffold0547 | 665279 | Non-genic |  | 0.7999 | 0.3201 | 0.1003 | 0.2689 |
|  | PgFD060 | Pg_scaffold0564 | 355366 | Non-genic |  | 0.8006 | 0.3193 | 0.0890 | 0.2683 |
|  | PgFD061 | Pg_scaffold0566 | 341954 | Non-genic |  | 0.6936 | 0.4251 | 0.1146 | 0.3347 |
|  | PgFD062 | Pg_scaffold0584 | 615603 | Non-genic |  | 0.8750 | 0.2188 | 0.0537 | 0.1948 |
|  | PgFD063 | Pg_scaffold0661 | 559200 | 5' 5kb | ID=Pg_S0661.4 | 0.6087 | 0.4764 | 0.1445 | 0.3629 |
|  | PgFD064 | Pg_scaffold0680 | 789693 | Non-genic |  | 0.9000 | 0.1800 | 0.0497 | 0.1638 |
|  | PgFD065 | Pg_scaffold0714 | 451183 | Non-genic |  | 0.7569 | 0.3680 | 0.1212 | 0.3003 |
|  | PgFD066 | Pg_scaffold0756 | 683302 | 5' 3kb | ID=Pg_S0756.9 | 0.9144 | 0.1566 | 0.0417 | 0.1444 |
|  | PgFD067 | Pg_scaffold0877 | 521064 | Non-genic |  | 0.8667 | 0.2311 | 0.0710 | 0.2044 |
|  | PgFD068 | Pg_scaffold0886 | 497950 | Exon | ID=Pg_S0886.4 | 0.9428 | 0.1079 | 0.0220 | 0.1021 |
|  | PgFD069 | Pg_scaffold0895 | 545059 | Intron | ID=Pg_S0895.33 | 0.9049 | 0.1721 | 0.0065 | 0.1573 |
|  | PgFD070 | Pg_scaffold0923 | 53986 | Non-genic |  | 0.5625 | 0.4922 | 0.1389 | 0.3711 |
|  | PgFD071 | Pg_scaffold0940 | 220865 | Non-genic |  | 0.7483 | 0.3767 | 0.1132 | 0.3058 |
|  | PgFD072 | Pg_scaffold1096 | 341342 | 5' 3kb | ID=Pg_S1096.7 | 0.7445 | 0.3804 | 0.1110 | 0.3081 |
|  | PgFD073 | Pg_scaffold1104 | 270437 | 5' 2kb | ID=Pg_S1104.15 | 0.9451 | 0.1038 | 0.0266 | 0.0984 |
|  | PgFD074 | Pg_scaffold1179 | 502863 | Non-genic |  | 0.9498 | 0.0954 | 0.0266 | 0.0909 |
|  | PgFD075 | Pg_scaffold1233 | 39706 | Exon | ID=Pg_S1233.6 | 0.6391 | 0.4613 | 0.1420 | 0.3549 |
|  | PgFD076 | Pg_scaffold1235 | 224820 | Non-genic |  | 0.6926 | 0.4258 | 0.1211 | 0.3351 |
|  | PgFD077 | Pg_scaffold1259 | 100166 | Non-genic |  | 0.5901 | 0.4838 | 0.1432 | 0.3668 |
|  | PgFD078 | Pg_scaffold1304 | 296892 | 3' 3kb | ID=Pg_S1304.21 | 0.5742 | 0.4890 | 0.1260 | 0.3694 |
|  | PgFD079 | Pg_scaffold1348 | 290326 | 5' 1kb | ID=Pg_S1348.17 | 0.7692 | 0.3551 | 0.1079 | 0.2920 |
|  | PgFD080 | Pg_scaffold1417 | 208866 | Non-genic |  | 0.6380 | 0.4619 | 0.1420 | 0.3552 |
|  | PgFD081 | Pg_scaffold1493 | 15961 | Non-genic |  | 0.6507 | 0.4546 | 0.1328 | 0.3513 |
|  | PgFD082 | Pg_scaffold1592 | 453567 | Non-genic |  | 0.5289 | 0.4983 | 0.1538 | 0.3742 |
|  | PgFD083 | Pg_scaffold1595 | 339159 | Non-genic |  | 0.5881 | 0.4845 | 0.1425 | 0.3671 |
|  | PgFD085 | Pg_scaffold1630 | 447308 | Non-genic |  | 0.6374 | 0.4622 | 0.1404 | 0.3554 |
|  | PgFD086 | Pg_scaffold1633 | 232248 | Non-genic |  | 0.9363 | 0.1192 | 0.0370 | 0.1121 |
|  | PgFD087 | Pg_scaffold1670 | 92895 | Non-genic |  | 0.9116 | 0.1612 | 0.0358 | 0.1482 |
|  | PgFD088 | Pg_scaffold1678 | 521819 | 3' 4kb | ID=Pg_S1678.56 | 0.9058 | 0.1707 | 0.0520 | 0.1561 |
|  | PgFD089 | Pg_scaffold1732 | 206920 | Intron | ID=Pg_S1732.3 | 0.7564 | 0.3685 | 0.0951 | 0.3006 |
|  | PgFD090 | Pg_scaffold1918 | 434648 | Exon | ID=Pg_S1918.5 | 0.6133 | 0.4743 | 0.1699 | 0.3618 |
|  | PgFD091 | Pg_scaffold1992 | 43720 | Non-genic |  | 0.7170 | 0.4058 | 0.1377 | 0.3235 |
|  | PgFD092 | Pg_scaffold2005 | 289838 | Non-genic |  | 0.5290 | 0.4983 | 0.1578 | 0.3742 |
|  | PgFD093 | Pg_scaffold2183 | 409208 | Non-genic |  | 0.9141 | 0.1571 | 0.0496 | 0.1448 |
|  | PgFD094 | Pg_scaffold2214 | 361068 | Non-genic |  | 0.8588 | 0.2425 | 0.0787 | 0.2131 |
|  | PgFD095 | Pg_scaffold2284 | 193556 | Non-genic |  | 0.6915 | 0.4267 | 0.1136 | 0.3357 |
|  | PgFD096 | Pg_scaffold2389 | 247078 | Non-genic |  | 0.6776 | 0.4369 | 0.1396 | 0.3415 |
|  | PgFD097 | Pg_scaffold2468 | 17877 | Non-genic |  | 0.5742 | 0.4890 | 0.1508 | 0.3694 |
|  | PgFD098 | Pg_scaffold2512 | 175225 | 5' 2kb | ID=Pg_S2512.4 | 0.8562 | 0.2462 | 0.0566 | 0.2159 |
|  | PgFD099 | Pg_scaffold2557 | 67635 | Non-genic |  | 0.5264 | 0.4986 | 0.1833 | 0.3743 |
|  | PgFD100 | Pg_scaffold2582 | 71450 | Non-genic |  | 0.9066 | 0.1694 | 0.0554 | 0.1550 |
|  | PgFD101 | Pg_scaffold2633 | 168672 | Exon | ID=Pg_S2633.5 | 0.7069 | 0.4144 | 0.1329 | 0.3285 |
|  | PgFD102 | Pg_scaffold2636 | 343928 | Exon | ID=Pg_S2636.25 | 0.8320 | 0.2796 | 0.0881 | 0.2405 |
|  | PgFD103 | Pg_scaffold2749 | 332850 | 3' 3kb | ID=Pg_S2749.21 | 0.7459 | 0.3790 | 0.1563 | 0.3072 |
|  | PgFD104 | Pg_scaffold2768 | 131879 | Non-genic |  | 0.9417 | 0.1098 | 0.0242 | 0.1038 |
|  | PgFD105 | Pg_scaffold3103 | 287542 | NA |  | 0.5700 | 0.4902 | 0.1673 | 0.3700 |
|  | PgFD106 | Pg_scaffold3125 | 186933 | 5' 5kb | ID=Pg_S3125.17 | 0.6786 | 0.4362 | 0.1387 | 0.3411 |
|  | PgFD107 | Pg_scaffold3141 | 147560 | Non-genic |  | 0.7297 | 0.3944 | 0.1192 | 0.3166 |
|  | PgFD108 | Pg_scaffold3192 | 220397 | Non-genic |  | 0.6672 | 0.4441 | 0.1280 | 0.3455 |
|  | PgFD109 | Pg_scaffold3238 | 9187 | Non-genic |  | 0.5755 | 0.4886 | 0.1350 | 0.3692 |
|  | PgFD110 | Pg_scaffold3258 | 59720 | Non-genic |  | 0.5954 | 0.4818 | 0.1364 | 0.3657 |
|  | PgFD111 | Pg_scaffold3443 | 178814 | Non-genic |  | 0.8480 | 0.2578 | 0.0566 | 0.2246 |
|  | PgFD112 | Pg_scaffold3527 | 86198 | Non-genic |  | 0.8737 | 0.2207 | 0.0450 | 0.1963 |
|  | PgFD113 | Pg_scaffold3565 | 33790 | 3' 1kb | ID=Pg_S3565.1 | 0.5266 | 0.4986 | 0.1574 | 0.3743 |
|  | PgFD114 | Pg_scaffold3741 | 130038 | Non-genic |  | 0.8626 | 0.2370 | 0.0675 | 0.2089 |
|  | PgFD115 | Pg_scaffold3822 | 17233 | Non-genic |  | 0.7658 | 0.3587 | 0.0984 | 0.2944 |
|  | PgFD116 | Pg_scaffold3932 | 145261 | NA |  | 0.8646 | 0.2342 | 0.1296 | 0.2067 |
|  | PgFD117 | Pg_scaffold4143 | 140262 | 3' 5kb | ID=Pg_S4143.5 | 0.9312 | 0.1281 | 0.0474 | 0.1199 |
|  | PgFD118 | Pg_scaffold4267 | 195742 | 3' 3kb | ID=Pg_S4267.18 | 0.7301 | 0.3941 | 0.0960 | 0.3165 |
|  | PgFD119 | Pg_scaffold4366 | 135643 | Non-genic |  | 0.9559 | 0.0842 | 0.0223 | 0.0807 |
|  | PgFD120 | Pg_scaffold4530 | 12646 | Intron | ID=Pg_S4530.1 | 0.5358 | 0.4974 | 0.1504 | 0.3737 |
|  | PgFD121 | Pg_scaffold4571 | 180274 | Non-genic |  | 0.8731 | 0.2216 | 0.0600 | 0.1970 |
|  | PgFD123 | Pg_scaffold5203 | 144050 | Intron | ID=Pg_S5203.4 | 0.7895 | 0.3324 | 0.1027 | 0.2771 |
|  | PgFD124 | Pg_scaffold5403 | 132667 | Intron | ID=Pg_S5403.7 | 0.8470 | 0.2592 | 0.0751 | 0.2256 |
|  | PgFD125 | Pg_scaffold5577 | 78461 | 5' 2kb | ID=Pg_S5577.13 | 0.8662 | 0.2318 | 0.0646 | 0.2049 |
|  | PgFD127 | Pg_scaffold6630 | 32554 | Non-genic |  | 0.7252 | 0.3986 | 0.1201 | 0.3192 |
|  | PgFD128 | Pg_scaffold6836 | 43762 | NA |  | 0.9688 | 0.0604 | 0.0185 | 0.0586 |
|  | PgFD129 | Pg_scaffold7492 | 52386 | Non-genic |  | 0.6495 | 0.4553 | 0.1236 | 0.3516 |
|  | PgFD130 | Pg_scaffold8025 | 10407 | Non-genic |  | 0.7416 | 0.3833 | 0.1080 | 0.3098 |
|  | PgFD131 | Pg_scaffold2156 | 250052 | Exon | ID=Pg_S2156.7 | 0.7329 | 0.3915 | 0.1161 | 0.3149 |
|  | PgFD132 | Pg_scaffold0429 | 625474 | 3' 1kb | ID=Pg_S0429.5 | 0.5254 | 0.4987 | 0.1607 | 0.3744 |
|  | PgFD133 | Pg_scaffold1005 | 229194 | 3' 1kb | ID=Pg_S1005.5 | 0.7171 | 0.4057 | 0.1085 | 0.3234 |
|  | PgFD134 | Pg_scaffold1335 | 196636 | Intron | ID=Pg_S1335.18 | 0.6806 | 0.4348 | 0.1134 | 0.3403 |
|  | PgFD135 | Pg_scaffold5398 | 77489 | Intron | ID=Pg_S5398.9 | 0.5711 | 0.4899 | 0.1249 | 0.3699 |
|  | PgFD136 | Pg_scaffold1400 | 260817 | Non-genic |  | 0.5242 | 0.4988 | 0.1386 | 0.3744 |
|  | PgFD137 | Pg_scaffold2132 | 449182 | 5' 3kb | ID=Pg_S2132.9 | 0.7793 | 0.3440 | 0.0962 | 0.2849 |
|  | PgFD138 | Pg_scaffold2932 | 138273 | 5' 4kb | ID=Pg_S2932.12 | 0.5949 | 0.4820 | 0.1435 | 0.3658 |
|  | PgFD140 | Pg_scaffold5500 | 94483 | 3' 3kb | ID=Pg_S5500.2 | 0.6447 | 0.4581 | 0.1343 | 0.3532 |
|  | pgFD151 | Pg_scaffold1697 | 246911 | Exon | ID=Pg_S1697.3 | 0.9653 | 0.0670 | 0.0162 | 0.0647 |
|  | pgFD152 | Pg_scaffold3220 | 193630 | Intron | ID=Pg_S3220.3 | 0.5823 | 0.4865 | 0.1263 | 0.3681 |
|  | pgFD153 | Pg_scaffold1128 | 343556 | Non-genic |  | 0.9454 | 0.1032 | 0.0223 | 0.0979 |
|  | pgFD154 | Pg_scaffold0460 | 896489 | Non-genic |  | 0.6807 | 0.4347 | 0.1010 | 0.3402 |
|  | pgFD155 | Pg_scaffold0272 | 1255141 | Intron | ID=Pg_S0272.6 | 0.9197 | 0.1478 | 0.0543 | 0.1369 |
|  | pgFD157 | Pg_scaffold1447 | 542014 | Non-genic |  | 0.8197 | 0.2956 | 0.0994 | 0.2519 |
|  | pgFD158 | Pg_scaffold6759 | 22413 | Non-genic |  | 0.7815 | 0.3415 | 0.0902 | 0.2832 |
|  | pgFD159 | Pg_scaffold1420 | 147004 | Non-genic |  | 0.9732 | 0.0522 | 0.0085 | 0.0509 |
|  | pgFD160 | Pg_scaffold3306 | 267108 | Non-genic |  | 0.8023 | 0.3172 | 0.0889 | 0.2669 |
|  | pgFD161 | Pg_scaffold1544 | 394595 | Non-genic |  | 0.8082 | 0.3100 | 0.0889 | 0.2620 |
|  | pgFD162 | Pg_scaffold3959 | 95794 | Non-genic |  | 0.8836 | 0.2057 | 0.0639 | 0.1846 |
|  | pgFD163 | Pg_scaffold1238 | 20658 | 5' 3kb | ID=Pg_S1238.3 | 0.5525 | 0.4945 | 0.1407 | 0.3722 |
|  | pgFD165 | Pg_scaffold1055 | 542398 | Non-genic |  | 0.5815 | 0.4867 | 0.1202 | 0.3683 |
|  | pgFD166 | Pg_scaffold8181 | 10903 | NA |  | 0.9383 | 0.1158 | 0.0334 | 0.1091 |
|  | pgFD167 | Pg_scaffold1508 | 253903 | Non-genic |  | 0.8826 | 0.2072 | 0.0526 | 0.1858 |
|  | pgFD168 | Pg_scaffold6754 | 6308 | NA |  | 0.9931 | 0.0138 | 0.0000 | 0.0137 |
|  | pgFD169 | Pg_scaffold3353 | 288408 | Non-genic |  | 0.9269 | 0.1355 | 0.0503 | 0.1263 |
|  | pgFD170 | Pg_scaffold1180 | 578136 | Non-genic |  | 0.5354 | 0.4975 | 0.1705 | 0.3737 |
|  | pgFD171 | Pg_scaffold2962 | 315059 | Non-genic |  | 0.7786 | 0.3447 | 0.1075 | 0.2853 |
|  | pgFD172 | Pg_scaffold1059 | 119856 | Intron | ID=Pg_S1059.10 | 0.8730 | 0.2218 | 0.0762 | 0.1972 |
|  | pgFD173 | Pg_scaffold1511 | 17849 | Non-genic |  | 0.8874 | 0.1998 | 0.0497 | 0.1799 |
|  | pgFD174 | Pg_scaffold0035 | 14657 | Intron | ID=Pg_S0035.16 | 0.8567 | 0.2455 | 0.0592 | 0.2154 |
|  | pgFD176 | Pg_scaffold4254 | 108939 | NA |  | 0.9642 | 0.0690 | 0.0115 | 0.0666 |
|  | pgFD177 | Pg_scaffold0035 | 1846877 | Non-genic |  | 0.9663 | 0.0652 | 0.0233 | 0.0630 |
|  | pgFD178 | Pg_scaffold1514 | 352676 | Non-genic |  | 0.9618 | 0.0736 | 0.0116 | 0.0708 |
|  | pgFD179 | Pg_scaffold2903 | 292911 | 5' 1kb | ID=Pg_S2903.25 | 0.8441 | 0.2632 | 0.0716 | 0.2285 |
|  | pgFD180 | Pg_scaffold0012 | 880466 | Non-genic |  | 0.9410 | 0.1110 | 0.0301 | 0.1048 |
|  | pgFD181 | Pg_scaffold4384 | 57080 | 5' 2kb | ID=Pg_S4384.2 | 0.9439 | 0.1059 | 0.0280 | 0.1003 |
|  | pgFD182 | Pg_scaffold5121 | 112210 | Exon | ID=Pg_S5121.6 | 0.7262 | 0.3977 | 0.0988 | 0.3186 |
|  | pgFD183 | Pg_scaffold0540 | 257856 | Non-genic |  | 0.5606 | 0.4927 | 0.1469 | 0.3713 |
|  | pgFD184 | Pg_scaffold1253 | 611520 | Non-genic |  | 0.9590 | 0.0786 | 0.0266 | 0.0755 |
|  | pgFD185 | Pg_scaffold2120 | 137707 | 5' 1kb | ID=Pg_S2120.11 | 0.8759 | 0.2174 | 0.0696 | 0.1938 |
|  | pgFD186 | Pg_scaffold1302 | 343001 | 5' 4kb | ID=Pg_S1302.21 | 0.8785 | 0.2135 | 0.0584 | 0.1907 |
|  | pgFD187 | Pg_scaffold2262 | 256184 | Non-genic |  | 0.9284 | 0.1329 | 0.0416 | 0.1241 |
|  | pgFD188 | Pg_scaffold6482 | 89758 | Intron | ID=Pg_S6482.5 | 0.9196 | 0.1479 | 0.0451 | 0.1370 |
|  | pgFD189 | Pg_scaffold7722 | 32962 | Exon | ID=Pg_S7722.3 | 0.6081 | 0.4766 | 0.1202 | 0.3630 |
|  | pgFD190 | Pg_scaffold5312 | 94911 | Intron | ID=Pg_S5312.3 | 0.9495 | 0.0958 | 0.0290 | 0.0912 |
|  | pgFD191 | Pg_scaffold1458 | 416094 | Non-genic |  | 0.9429 | 0.1077 | 0.0265 | 0.1019 |
|  | pgFD192 | Pg_scaffold1510 | 93824 | Non-genic |  | 0.8632 | 0.2362 | 0.0543 | 0.2083 |
|  | pgFD193 | Pg_scaffold0180 | 1165104 | Non-genic |  | 0.9413 | 0.1105 | 0.0384 | 0.1044 |
|  | pgFD194 | Pg_scaffold1141 | 328721 | Non-genic |  | 0.8870 | 0.2004 | 0.0660 | 0.1803 |
|  | pgFD195 | Pg_scaffold0249 | 550285 | Non-genic |  | 0.9278 | 0.1339 | 0.0450 | 0.1250 |
|  | pgFD196 | Pg_scaffold0117 | 1339397 | Non-genic |  | 0.9163 | 0.1534 | 0.0497 | 0.1417 |
|  | pgFD197 | Pg_scaffold3096 | 300109 | Non-genic |  | 0.7856 | 0.3368 | 0.1086 | 0.2801 |
|  | pgFD198 | Pg_scaffold1277 | 427865 | Non-genic |  | 0.9266 | 0.1360 | 0.0405 | 0.1268 |
|  | pgFD199 | Pg_scaffold0675 | 866418 | Non-genic |  | 0.9236 | 0.1411 | 0.0423 | 0.1311 |
|  | pgFD200 | Pg_scaffold1506 | 58845 | Non-genic |  | 0.9186 | 0.1496 | 0.0358 | 0.1384 |
|  | pgFD201 | Pg_scaffold1506 | 58909 | Non-genic |  | 0.6845 | 0.4319 | 0.1327 | 0.3386 |
|  | pgFD202 | Pg_scaffold2786 | 88577 | 3' 3kb | ID=Pg_S2786.1 | 0.9641 | 0.0693 | 0.0271 | 0.0669 |
|  | pgFD203 | Pg_scaffold0923 | 283506 | Non-genic |  | 0.5261 | 0.4986 | 0.1417 | 0.3743 |
|  | pgFD204 | Pg_scaffold1672 | 192701 | Non-genic |  | 0.9333 | 0.1245 | 0.0360 | 0.1168 |
|  | pgFD205 | Pg_scaffold1451 | 396204 | 5' 4kb | ID=Pg_S1451.4 | 0.8830 | 0.2066 | 0.0757 | 0.1853 |
|  | pgFD206 | Pg_scaffold0461 | 889437 | Non-genic |  | 0.5376 | 0.4972 | 0.1457 | 0.3736 |
|  | pgFD207 | Pg_scaffold1441 | 388694 | Non-genic |  | 0.5803 | 0.4871 | 0.1572 | 0.3685 |
|  | pgFD208 | Pg_scaffold0057 | 1923400 | Non-genic |  | 0.9459 | 0.1023 | 0.0221 | 0.0971 |
|  | pgFD209 | Pg_scaffold0001 | 3132955 | Non-genic |  | 0.9221 | 0.1436 | 0.0473 | 0.1333 |
|  | pgFD211 | Pg_scaffold0917 | 459327 | 3' 2kb | ID=Pg_S0917.35 | 0.7137 | 0.4087 | 0.1220 | 0.3252 |
|  | pgFD213 | Pg_scaffold2946 | 90555 | 3' 1kb | ID=Pg_S2946.7 | 0.9588 | 0.0791 | 0.0212 | 0.0759 |
|  | pgFD214 | Pg_scaffold1244 | 442097 | Non-genic |  | 0.8387 | 0.2705 | 0.0682 | 0.2339 |
|  | pgFD215 | Pg_scaffold0735 | 616311 | Non-genic |  | 0.8486 | 0.2570 | 0.0740 | 0.2240 |
|  | pgFD216 | Pg_scaffold2974 | 222938 | Non-genic |  | 0.8559 | 0.2467 | 0.0618 | 0.2163 |
| Plastid | PgFD141 |  | 21344 | CDS | rpoC2 | 0.8043 | 0.3148 | 0.0035 | 0.2653 |
|  | PgFD142 |  | 117376 | CDS | ccsA | 0.9342 | 0.1230 | 0.0000 | 0.1154 |
|  | PgFD144 |  | 22287 | CDS | rpoC1 | 0.9118 | 0.1609 | 0.0012 | 0.1480 |
|  | pgFD145 |  | 7159 | CDS | rps16 ~ trnQ-UUG | 0.9931 | 0.0137 | 0.0000 | 0.0137 |
|  | pgFD147 |  | 44895 | Intron | ycf3 | 0.9827 | 0.0340 | 0.0000 | 0.0334 |
|  | pgFD149 |  | 118525 | CDS | ndhD | 0.9625 | 0.0722 | 0.0012 | 0.0696 |
| Mean |  |  |  |  |  | 0.7787 | 0.3035 | 0.0903 | 0.2464 |

^a^ Major allele frequency

^b^ Gene diversity

^c^ Heterozygosity

^d^ Polymorphism information content

**Table S6.** Information of 192 Fluidigm SNP chip assay developed in this study.

| **Marker** | **FAM** | **HEX** | **ASP primer sequence (5' → 3')** | | **LSP primer sequence (5' → 3')** | **STA primer sequence (5' → 3')** |
| --- | --- | --- | --- | --- | --- | --- |
|  |  |  |  |  |  |  |
| PgFD001 | T | C | F ^a^ | CGAGCACTCCCTCCTCTTCT | GCCGGGAGGTTTTTGGACA | TCTCTTCCTCCACCTGGC |
|  |  |  | H ^b^ | GAGCACTCCCTCCTCTTCC |  |  |
| PgFD002 | A | T | F | GCCTTCTGAATTCTCGGAGCA | GTGAGCTCGTTTGGCAGTTCA | GACCTTACTATCACCAAAACCTCTG |
|  |  |  | H | GCCTTCTGAATTCTCGGAGCT |  |  |
| PgFD003 | A | C | F | TGCAGCAGAATTCAAAGAATGCAAA | CTGCGCTCCCTTCCTTTGT | TGAGTGTGTGGACTGAAGCT |
|  |  |  | H | GCAGCAGAATTCAAAGAATGCAAC |  |  |
| PgFD005 | T | C | F | ACAAAGTTAACCCAGTGTCTAGCA | AACCTGCCAGGATGACCCA | TGATTGAGGATTTACAAAGACAAGTGA |
|  |  |  | H | ACAAAGTTAACCCAGTGTCTAGCG |  |  |
| PgFD006 | A | T | F | TCTAACAAGAAGAGTGGGATGCATAA | GAACACCCCTACTTCCCCCT | CTTTGGAGACATTGCGCGT |
|  |  |  | H | TCTAACAAGAAGAGTGGGATGCATAT |  |  |
| PgFD007 | A | G | F | CATGACCTTTCTATAGGAGCTACGTAA | CGGCCCCTATAAGACTCAGCA | TGCAGTAGGGAGGCTTAGAC |
|  |  |  | H | CATGACCTTTCTATAGGAGCTACGTAG |  |  |
| PgFD008 | G | A | F | GCTTGCTTGATACTGGGCTG | AGCAGCTTTTACCTAGCCTCACA | GGTTTGTCTAGGCTGCAAGA |
|  |  |  | H | TGCTTGCTTGATACTGGGCTA |  |  |
| PgFD009 | A | T | F | CCAGGGCACTATGCTCATTCA | GGACCCAGTGCCCCGA | TGGTTTTGCCTGCATTTAGAGTG |
|  |  |  | H | CCAGGGCACTATGCTCATTCT |  |  |
| PgFD010 | T | C | F | GGAATATTCTGTCGGAATTAGGCCTTT | CCCGAATCGGAACGTCCACA | TCGTGGGAATAGGATGTTATCTGAA |
|  |  |  | H | GAATATTCTGTCGGAATTAGGCCTTC |  |  |
| PgFD011 | A | C | F | GATTTACTGGGGTGAATTCATGTGAA | GAATCCGCCCCGACGGATA | CGAATTGGTTTCCTTGGTTGG |
|  |  |  | H | GATTTACTGGGGTGAATTCATGTGAC |  |  |
| PgFD012 | G | A | F | TCCTCCACCAAGAAAGTCTTGAG | TCGATCAAGAATTTGTGGGAACCGA | GAAGGGATTTCCAGAATTCCTCC |
|  |  |  | H | TCCTCCACCAAGAAAGTCTTGAA |  |  |
| PgFD014 | C | T | F | AAGCTCCTTCTTCCTGCCC | TCATCTTGCAGAGGCACACGA | GCCACTAACACCGAAGTTGATAAA |
|  |  |  | H | CAAGCTCCTTCTTCCTGCCT |  |  |
| PgFD016 | A | G | F | TCATCTATGCATTTCTCAGTCTCGTT | CAATTGGGCGAACCCCTGG | AGGTAAGATTCCTCCTTCACGAAT |
|  |  |  | H | TCATCTATGCATTTCTCAGTCTCGTC |  |  |
| PgFD017 | A | T | F | CCCAAGGATCAACTCAATGTCTTATAACA | CCCTTGGCTCTCCCTATGGA | ACCCAATCTTTACCAATCTCTCTGA |
|  |  |  | H | CCCAAGGATCAACTCAATGTCTTATAACT |  |  |
| PgFD018 | C | T | F | CGCCTTCATGGCAGATGG | GGTTCGCAAAATTAGTGTGGAACTCA | GTCGATTGATGTCTGAACTCCG |
|  |  |  | H | CCGCCTTCATGGCAGATGA |  |  |
| PgFD020 | A | T | F | ATTACACAAAACTGCAAAAAAATTCTGAATTCTAT | CCCGAACTGGCACTGCG | GAGTTAGAAAAGTTAGCGTCAGAATCA |
|  |  |  | H | ATTACACAAAACTGCAAAAAAATTCTGAATTCTAA |  |  |
| PgFD022 | A | G | F | GGATGAGGAGGCAACAAGTTTTTTT | GCGCCATCTGCCTCACAT | TGAGGATGAGGAGGCAACA |
|  |  |  | H | GGATGAGGAGGCAACAAGTTTTTTC |  |  |
| PgFD024 | T | C | F | GCACATTGCTATAAAATCCTACCATCCT | CAAAATTCTGCAGATATATAATGGATTTCATCACGA | ACCACAGAGAATATCATACATCGCA |
|  |  |  | H | CACATTGCTATAAAATCCTACCATCCC |  |  |
| PgFD025 | C | T | F | CAGCGAGTTACTCTTTGCCTG | CCGGGGTAAATTCGTTGGCA | CCGACTGATTCTAAATTCGCCC |
|  |  |  | H | CCAGCGAGTTACTCTTTGCCTA |  |  |
| PgFD026 | A | C | F | GTTTTGATTCCTTCGCTCTTGCAT | TGCTCCGGACAGACTGCG | CGAACAATCCGCTGCGT |
|  |  |  | H | TTTTGATTCCTTCGCTCTTGCAG |  |  |
| PgFD027 | A | G | F | AGACCAAACAACACCTACTTTCCT | GCTCCATTCCTCGCATTCGT | GGGCCCTCACATTATAAAACTCAT |
|  |  |  | H | GACCAAACAACACCTACTTTCCC |  |  |
| PgFD028 | A | G | F | GAAGCTAGTTCACCCTATCCACA | CTCTCTTGTAGCAACTGTGCCC | CTCCTGTACTAGAAGCTCTAGGG |
|  |  |  | H | GAAGCTAGTTCACCCTATCCACG |  |  |
| PgFD029 | C | T | F | GGTGCTAAGGAATTCTCTCAAAGC | CACTTGAGTGGGCGCCG | TGAAATAGTTCAATTACAGCGCCT |
|  |  |  | H | TGGTGCTAAGGAATTCTCTCAAAGT |  |  |
| PgFD030 | A | G | F | CGTATGGACTAAAAATGTTTGGTGTTGT | CCGCACTAACACACTGCCC | GGTTGAATTCAGCTGATTCCCG |
|  |  |  | H | GTATGGACTAAAAATGTTTGGTGTTGC |  |  |
| PgFD031 | C | T | F | CATGTTCAAAAAAGGATCGCAAAGC | CTCGGGGATCGCTGTCGT | GCAAAAGTGAATTTCGCCATGA |
|  |  |  | H | ACATGTTCAAAAAAGGATCGCAAAGT |  |  |
| PgFD032 | C | T | F | GTTGAATTCAGGGTTTCGACCG | CCCCACTCCTCGCCGT | GTCGATATCAACATGATTACTATATAGCCAT |
|  |  |  | H | AGTTGAATTCAGGGTTTCGACCA |  |  |
| PgFD033 | C | T | F | TCAGTTCATGGCAGAAGGCC | GCACAACTTGATGTCGAACTCCT | ACTGAACAGAGTGAGAAAGAAGTCT |
|  |  |  | H | TCAGTTCATGGCAGAAGGCT |  |  |
| PgFD034 | G | A | F | GGAGTCGAATTCGATGGTTTCAG | GAGTGCTCATCACTCGACCCA | AGACATGAAGATTTGGAACTCATCATT |
|  |  |  | H | GGAGTCGAATTCGATGGTTTCAA |  |  |
| PgFD035 | C | T | F | CTTTCAAGACTACAGATAGTCCCCAG | ACATCTGCATCACTTCAGCCGA | CCCTAACCGATGAGCTAAGATTTTA |
|  |  |  | H | CTTTCAAGACTACAGATAGTCCCCAA |  |  |
| PgFD038 | T | C | F | CTCCTCCCCCTTATTCCCAT | CAAGTGCGTTCAAAAGTAGTTGGGT | TGACCTTTCTTCCTCCTCCC |
|  |  |  | H | CTCCTCCCCCTTATTCCCAC |  |  |
| PgFD039 | C | T | F | GCGCAGAAAAGGTCCTACTAGC | GCAGGTCCTATTAGCGGCAAG | CCTGCAGTCAATATGAAAAGGTCA |
|  |  |  | H | GCGCAGAAAAGGTCCTACTAGT |  |  |
| PgFD040 | C | T | F | GGCATCTGGGCTGAATGAAG | CAAAGCGTTTGTGCGGGGTA | GGCACGCAAGCTATAGTACTCA |
|  |  |  | H | TGGCATCTGGGCTGAATGAAA |  |  |
| PgFD043 | T | C | F | GCAAAAACTTGAGAAAGTCGGCA | TCAGTCACGGTCATTGCCCA | ACGTTCGACTTCTACTTCTAGTTCT |
|  |  |  | H | GCAAAAACTTGAGAAAGTCGGCG |  |  |
| PgFD045 | G | A | F | GCATGTTTCGAAAGTCTTGTTTGGG | GGAGTTTTTCGGTCTTACGACTAGACT | GGGTCACGGATCGCATG |
|  |  |  | H | GCATGTTTCGAAAGTCTTGTTTGGA |  |  |
| PgFD046 | C | T | F | GTTGCTGGTGTGCAGACAG | AGGTTGCTGATAGTGGTCGCA | CGGACATGTCGTTCGCTAAAATAA |
|  |  |  | H | GTTGCTGGTGTGCAGACAA |  |  |
| PgFD047 | A | T | F | AGCTTCAACAGTTCATATGTCAAAAGAAATAAT | GGAAGCATAGCATTTCATTTCAGTCTACTTT | ACACAGTTGTCAAATATAAAAGCTTCAAC |
|  |  |  | H | AGCTTCAACAGTTCATATGTCAAAAGAAATAAA |  |  |
| PgFD048 | A | T | F | GAAGGCATACAAAAGGGCAAAAAAA | ACGGATTCAAGCTCCCCGT | CTCCGCAAGACTTGTCGAA |
|  |  |  | H | GAAGGCATACAAAAGGGCAAAAAAT |  |  |
| PgFD049 | A | G | F | AAGAGGTTTTAGCTCTAGCACCAA | CTGGCCTGAAGGAGGAGACA | CCAAGTATGCATACCGTAAATGAGA |
|  |  |  | H | AAGAGGTTTTAGCTCTAGCACCAG |  |  |
| PgFD050 | C | T | F | AAGATTAAGTCCGAGAGTTATAGGACTTTTC | CCTTTCTGGTATTTCATACACATCTCTCTATTACT | GCAAGTGCAAAGTTGAAAAGTGTAA |
|  |  |  | H | AAGATTAAGTCCGAGAGTTATAGGACTTTTT |  |  |
| PgFD051 | T | C | F | CCCAACTTGCCCAGCCA | AACTGGGTTTTTGAGTTTGGCGA | GCCTCCTCAATTCTCTCTGTCTAG |
|  |  |  | H | CCAACTTGCCCAGCCG |  |  |
| PgFD052 | T | C | F | TTCAAATTACAGACATGAAAGCTGAAGAAA | AACTCTCTCCCAATTCCCAACCA | TCAGCTCCAGCTATAAATCCGT |
|  |  |  | H | TTCAAATTACAGACATGAAAGCTGAAGAAG |  |  |
| PgFD053 | G | A | F | ACAAATGCATGACTTGGCTAACTTG | TGCCCCAACCCCTCGT | TCGGTATTGGAACGGATCAGA |
|  |  |  | H | GACAAATGCATGACTTGGCTAACTTA |  |  |
| PgFD054 | C | T | F | CTTGTTTCAGAGTTGTTCCGCG | CCCACTCGAAACCAGGGAACT | CCAAAAGAAATCATAGAATCGTGTCG |
|  |  |  | H | CTTGTTTCAGAGTTGTTCCGCA |  |  |
| PgFD055 | A | T | F | CTGGGAAGGTTTTGTAAATGAGCT | GCCCAAAAAGCATCAACTATGCTGG | CGCTTCAACTTTCTCTAGAAGCATT |
|  |  |  | H | CTGGGAAGGTTTTGTAAATGAGCA |  |  |
| PgFD056 | A | T | F | GCTGAAGTGGATTGGGAAACTTTTA | TGCCATGTATATAAATGCATGCTAAGAAGAGA | TCTGTTTGCTGACTTTTCCTGTG |
|  |  |  | H | GCTGAAGTGGATTGGGAAACTTTTT |  |  |
| PgFD057 | A | G | F | AGATGCATGCCTTTTGTCAACTTTT | GGGACAACACTATTTCGTAAATGTGCT | GCACATAAACAAATATTAGATGCATGCC |
|  |  |  | H | AGATGCATGCCTTTTGTCAACTTTC |  |  |
| PgFD058 | T | C | F | CCTGTCCAGAAACCTGTTTGGT | CCACTCTAGGGCAAAGATTCTCCT | AGTTGCTATGCATAGACTTTGTGG |
|  |  |  | H | CTGTCCAGAAACCTGTTTGGC |  |  |
| PgFD059 | A | G | F | CTTGAATAACATTTCTAAAATGCTCATAAGTGTTT | ATTTTCAACATTGGTTTAAAAGAAGGGTTGTAGA | AACTTATGAGTTTGAAGGGACCTTG |
|  |  |  | H | CTTGAATAACATTTCTAAAATGCTCATAAGTGTTC |  |  |
| PgFD060 | C | T | F | ACATGGAGTTATATAGCCAAGCTTTCTC | GGGCCAACCTAGTGTGAACG | AGTCTTAGGCAGACAGTTAAGGA |
|  |  |  | H | ACATGGAGTTATATAGCCAAGCTTTCTT |  |  |
| PgFD061 | G | A | F | CTAGCTCTGTACTAGCTAATTTTTGTTGC | GTTGGCTAAGGTAGGAGATCAGCA | ATGCCTGCCCCGGTG |
|  |  |  | H | TCTAGCTCTGTACTAGCTAATTTTTGTTGT |  |  |
| PgFD062 | G | A | F | CCACTCCCTTCATTAATGGCC | CGCCAAGGCTAAACCCAACA | TCTCGACGATGTAGCGCC |
|  |  |  | H | GCCACTCCCTTCATTAATGGCT |  |  |
| PgFD063 | T | G | F | TCCTGATCCTCTTGGGTACGA | GGGTGCCCCATCCCGA | CTGCCTGGTGTCCCGA |
|  |  |  | H | CCTGATCCTCTTGGGTACGC |  |  |
| PgFD064 | C | G | F | GGAGCAACGATGTCGCG | AGGAATTCTGGGTTCTGGGCA | GCGCTGCAACGCTTGG |
|  |  |  | H | GGAGCAACGATGTCGCC |  |  |
| PgFD065 | G | C | F | GTGGACACTAAGCTATCATCTGATGTATC | GAGCAGGAGAATATATCATGTTATTAAGTTTACTAACAAT | GCATGAGATGTAACTCATTAGGCT |
|  |  |  | H | TGGACACTAAGCTATCATCTGATGTATG |  |  |
| PgFD066 | G | A | F | GCAGGCCAATTCAAGCCAG | GACCAGATCAGCCAATGCCTT | CCGCTGGGATGTGGTTT |
|  |  |  | H | GCAGGCCAATTCAAGCCAA |  |  |
| PgFD067 | G | A | F | TGGCCGTGCCTCAGC | TTTTATGTCTCTTCTATGCATGGACCAATTTTTTT | CACATACGTACTGTGCCCTT |
|  |  |  | H | TTGGCCGTGCCTCAGT |  |  |
| PgFD068 | A | G | F | GCTTCCAAGCCTTCTTCCACA | AGGTTTATCTCGTTGGTGATATCGTAATCAA | CAGTGCCTATAGAATGAAGAGAAGC |
|  |  |  | H | CTTCCAAGCCTTCTTCCACG |  |  |
| PgFD069 | A | T | F | ACCTTTCGACACCTTGCTTGT | ATATATTCAATGTAAATTAATAACGAACATGTACGTACGG | TCATTGGTACATGTTCAACTCATTCA |
|  |  |  | H | ACCTTTCGACACCTTGCTTGA |  |  |
| PgFD070 | A | G | F | TCGAACTCTCTCTACTTCCACACTA | AGTTAACTTTCACCCTACAAATTCTCAGAATCA | AACCCATTTTCTTTATTCTCGGCT |
|  |  |  | H | CGAACTCTCTCTACTTCCACACTG |  |  |
| PgFD071 | A | G | F | GCCTTAGAAAGAGACCCAAAAACATTAT | GGGCAGTGATTCAGATGATGCA | CCTCTTCTTCTTCACTGGCCT |
|  |  |  | H | GCCTTAGAAAGAGACCCAAAAACATTAC |  |  |
| PgFD072 | A | G | F | GTAGCTCACTACACGCAGCA | CATTAGTAAGAATTCTCTTGGACCTCTGACA | GCCGAAGATATCGAGCGATG |
|  |  |  | H | AGCTCACTACACGCAGCG |  |  |
| PgFD073 | C | G | F | CAATAACTAAATTCTACACGAAAAGCAAAACG | TCATATTCTTTCAGCAATAATCTTTTTAATTTTGATTTGT | ACACTAATTTGAGAAAGAACAATCAGGT |
|  |  |  | H | CAATAACTAAATTCTACACGAAAAGCAAAACC |  |  |
| PgFD074 | G | A | F | AGAAGCTTCAGGGCATGTCC | CGAAGAGGCTTTAGCGCCG | CTTTTCGCGCCCCGAC |
|  |  |  | H | ATTAGAAGCTTCAGGGCATGTCT |  |  |
| PgFD075 | A | G | F | GATGCTAGTTGCAATTGATTGTTACCA | TCTGTGCTCCAACAAGGCCA | AGCTTGTTTGATACCCAAAAATTTTAAGAG |
|  |  |  | H | TGCTAGTTGCAATTGATTGTTACCG |  |  |
| PgFD076 | C | T | F | TCCATCCTTATGTGCACCCAG | TCGTGTAAAGGTGGGGCCA | GCAAAGAAAATGGTCCAGCGAG |
|  |  |  | H | TCCATCCTTATGTGCACCCAA |  |  |
| PgFD077 | A | G | F | GCTAAAATTCATACTTTGAATGCATGTATAGATGT | CTGGAACAATAACAAATAAAAAATTGAAAGTATCAGAAAA | AAAAACAGAGACTTGACAGATTTAGGTT |
|  |  |  | H | CTAAAATTCATACTTTGAATGCATGTATAGATGC |  |  |
| PgFD078 | A | C | F | ACCAATCTCTCCCCGTTAGGA | ACCAAAGAGTATGTTTAGGCTTTGCAC | ATCACCAATCTCTCCCCGTT |
|  |  |  | H | CCAATCTCTCCCCGTTAGGC |  |  |
| PgFD079 | A | C | F | GAGACTTGGAGTCAAGTTGTCCT | TCTTGATGGTCTCAAAAATATAATTATATACCACACAAAT | ACATATAGTTCCTTGGGGATGTATGTA |
|  |  |  | H | AGACTTGGAGTCAAGTTGTCCG |  |  |
| PgFD080 | T | G | F | CATACAGTAGGGCAGAGAATTTCATTT | CCCTGGCTTCAAACTCCCC | CCAAAAGCCTTGTTAGAGGCA |
|  |  |  | H | CATACAGTAGGGCAGAGAATTTCATTG |  |  |
| PgFD081 | G | T | F | GATGATGATGTGGATGCATCGAC | ACCGTGCCTTAGGTTACAATCCA | CAGTTATGAAGATGATCATGATTCGGA |
|  |  |  | H | TGATGATGATGTGGATGCATCGAA |  |  |
| PgFD082 | C | T | F | TGTTCCAACGTCGTTCAAAAATCTG | CGAGCATGGCCTTCATCCG | GGTCTGTTGGTCTGTGAAAGT |
|  |  |  | H | GTGTTCCAACGTCGTTCAAAAATCTA |  |  |
| PgFD083 | C | A | F | TTCCTCGTCAACCAGTCCATG | CCAGATGTGCTTGCTCGCAT | TCTAAAAGGGAAAGGCATAGATCTCA |
|  |  |  | H | TTCCTCGTCAACCAGTCCATT |  |  |
| PgFD085 | T | A | F | CAAGTTTGTAAGCACGAGTTGATCAA | CCATCAATGATGCATCATTTTCAAACACAAT | AGACCAACTTTGCATAGAAAGTCAA |
|  |  |  | H | CAAGTTTGTAAGCACGAGTTGATCAT |  |  |
| PgFD086 | G | T | F | CCCTCAAAGAGCAAACCACTG | GCATCTTTTTATGGACCATCTTGATATTGGAT | AGGAAGGATAATGTGATCATCCCT |
|  |  |  | H | TCCCTCAAAGAGCAAACCACTT |  |  |
| PgFD087 | G | A | F | GGCGGTGTTTCGTCCTTTTATTTC | GCCAAATCCTCATAACTCGGAACTTTC | GGCTGCTGATGTTCCTGAT |
|  |  |  | H | GGCGGTGTTTCGTCCTTTTATTTT |  |  |
| PgFD088 | G | A | F | TATATTGATCAATTGATCAACGTATTATGAAGTTAG | TCCGTAGAATATGACAAATCAGCTCTAACATTT | ATCTTTCTCTGCGTGTCAACATT |
|  |  |  | H | TATATTGATCAATTGATCAACGTATTATGAAGTTAA |  |  |
| PgFD089 | A | G | F | CGGGCTTTGACTAGGCCA | GCACAAAAGTCCCGCGACC | GGTCCAGGTCTTGACTAGGT |
|  |  |  | H | CGGGCTTTGACTAGGCCG |  |  |
| PgFD090 | C | T | F | ACTGGTTAGGAATCACCAATCCC | CAGGTCAATAATGCTACTGGCCC | TGAAGAAGAGGGTCCATCTCAAA |
|  |  |  | H | AACTGGTTAGGAATCACCAATCCT |  |  |
| PgFD091 | G | T | F | GTACAGTTTGGGTTAATGTGTCCC | GAACGAGTACCAGTTAGACATTATAGGTGT | ACTGACCTTGGATGACTTAGTACA |
|  |  |  | H | AGTACAGTTTGGGTTAATGTGTCCA |  |  |
| PgFD092 | A | C | F | CCTGTAGCAGCAGTTGGGATT | AGCAGAGCCCTGCCCA | TGGGATGGTTTATACTGCACCT |
|  |  |  | H | CCTGTAGCAGCAGTTGGGATG |  |  |
| PgFD093 | G | T | F | TCCAATTCGTCTGACTGATGACAG | CCCAAATAGGCATGTCCCCCT | CCGCCCAATATGCTTTGTGT |
|  |  |  | H | TCCAATTCGTCTGACTGATGACAT |  |  |
| PgFD094 | T | C | F | AAGGCTAGGAATTTATGGGCGT | GCATACCTTCAGCGCCCC | TGCAGAATCTTGAGAACAGCCTA |
|  |  |  | H | GGCTAGGAATTTATGGGCGC |  |  |
| PgFD095 | T | G | F | CCAAAATTCTGAAGGAAGGTGAACAT | CGCTTTCTCTCCACGAGGACT | CAATGGCTTCCAAATCTTCAACC |
|  |  |  | H | CCAAAATTCTGAAGGAAGGTGAACAG |  |  |
| PgFD096 | T | A | F | CGTAGATGACTTTTCCCACGTCAATA | GCATGTCTTCTGTTTGCCATTGTCA | TCAATGCATGGAGGATCGACT |
|  |  |  | H | GTAGATGACTTTTCCCACGTCAATT |  |  |
| PgFD097 | A | C | F | CGTAGAAGGAACAAGTAAAGTGCAAT | TGCTTCCTCACGTTATATGGATTGCT | CATGATGATAATGAATGCTCCACAC |
|  |  |  | H | CGTAGAAGGAACAAGTAAAGTGCAAG |  |  |
| PgFD098 | G | A | F | CATTGAGCCATGAAGGGCTC | CCGATGCTCACCCCTATAAGAAATATGC | CCAAGATCTCATGCATCCATATCTT |
|  |  |  | H | CATTGAGCCATGAAGGGCTT |  |  |
| PgFD099 | T | C | F | GGAAAGCCAGCGACCCAA | AGGCTCCTCGGCAGCA | GCCCATAAGCCTCAACTGC |
|  |  |  | H | GGAAAGCCAGCGACCCAG |  |  |
| PgFD100 | C | T | F | CGCGCTATTAGGAAGCTTTTTTCTG | TGGAATCCTGGTGCGCCT | TCTGTCCACATAGTGCGGG |
|  |  |  | H | CGCGCTATTAGGAAGCTTTTTTCTA |  |  |
| PgFD101 | C | T | F | GGTATTCGGCCCTTGCAAC | CCACGCCCATGGTTTGTCTT | CTATTACCGACGTTTCATTACGGG |
|  |  |  | H | GGTATTCGGCCCTTGCAAT |  |  |
| PgFD102 | C | T | F | GTTTCCTCGACGATGCATGAC | AGCTGGGAACCCACCCAA | CAAAGTCCAACTAGTCCAGTATCTG |
|  |  |  | H | TGTTTCCTCGACGATGCATGAT |  |  |
| PgFD103 | G | C | F | TTCAGGCTTGAACAATTCATCGAAC | GATTAGACCAGTGACCTATTTACCCGA | GCATGATGCATGCATAAACAATGT |
|  |  |  | H | TTTCAGGCTTGAACAATTCATCGAAG |  |  |
| PgFD104 | G | A | F | GGCGGTGCCTCAGATTGTAC | ACTGCGGTGCGAGACCA | CACCTCAAAACACCCTAACCTAC |
|  |  |  | H | GGCGGTGCCTCAGATTGTAT |  |  |
| PgFD105 | G | A | F | TCATCTCCTAAGGTCAAATCATGGATTG | ATGCATAGGTTCAAAGAGGTTCAAATTTGT | CACATCCTAGCTAAAGTCAGATTGAA |
|  |  |  | H | GTCATCTCCTAAGGTCAAATCATGGATTA |  |  |
| PgFD106 | G | A | F | AGGTAGCACCATGGACAAGTG | GCTTTGCCAACTAGCCCACA | TGTGTTGTGCATATTCTTCGCA |
|  |  |  | H | CAGGTAGCACCATGGACAAGTA |  |  |
| PgFD107 | C | T | F | GTTGTCCGGGACAACAAATAAAAAC | TGAACTTTTTTGTATACAACTTGTACTTTTTATTTATTTA | GCGGTTCATGAAATTTTGGTTGT |
|  |  |  | H | GGTTGTCCGGGACAACAAATAAAAAT |  |  |
| PgFD108 | C | G | F | ATTTCTACTGCGCATGATGGATAAAAC | TGCATATAAACTTTTGACCAAACAAATTCCATCT | TGTCATTAGATTTCTACTGCGCATG |
|  |  |  | H | ATTTCTACTGCGCATGATGGATAAAAG |  |  |
| PgFD109 | G | A | F | GAAACTTAGCAGAGAATAAGAAGTGGC | CTTGTGTGCATGCATGGCAGA | TCACCGTCATTGTACCTTAAAAAGAG |
|  |  |  | H | AGAAACTTAGCAGAGAATAAGAAGTGGT |  |  |
| PgFD110 | T | A | F | GTCAGAGAGTTGTCTCTGATCGT | GGATTGAAGATGAATCTCCAAATTAACCCA | TCGAAGTGGAATAGATCACTTGTCA |
|  |  |  | H | TGTCAGAGAGTTGTCTCTGATCGA |  |  |
| PgFD111 | T | C | F | GCCTTCATGGCAGATGGAGATA | GGTTCGCAAAATTAGTGTGGAACTCA | GTCGATTGATGTCTGAACTCCG |
|  |  |  | H | CCTTCATGGCAGATGGAGATG |  |  |
| PgFD112 | C | T | F | GATGTAGGCCCCCGCC | ACGCACAACGCACATGCATA | CTCACATAATACTCACACACACATGTA |
|  |  |  | H | GATGTAGGCCCCCGCT |  |  |
| PgFD113 | A | G | F | TGATTAGTTCTGACTATGCAGTAAGTTGTT | TGATGTTTGAGCATGCGATTGGTT | CTGACAGGAGAGAACTTATATGTGC |
|  |  |  | H | TGATTAGTTCTGACTATGCAGTAAGTTGTC |  |  |
| PgFD114 | G | A | F | CAGAGCTTTGTTGTCAACTTTGAAAG | GGGACGATCCAACAATGGCAA | GAACAACGTGTGATGCATTTTCC |
|  |  |  | H | CCAGAGCTTTGTTGTCAACTTTGAAAA |  |  |
| PgFD115 | A | G | F | GCTGATCGAGCCCATTGGAT | CCCAGAGGACTCAGATGTCCA | CTGAAGGCCTGGGATAAGTTT |
|  |  |  | H | GCTGATCGAGCCCATTGGAC |  |  |
| PgFD116 | C | T | F | CAATTGGGGGTTACGTGTATCG | GGGCCTTTGCCCAGATGATAGA | CCTAGATAAGAGCTATTAGAGCTGCA |
|  |  |  | H | GCAATTGGGGGTTACGTGTATCA |  |  |
| PgFD117 | T | G | F | AGAGAAACAGACTTTCATGTGCTCA | GGAGAAAACACACAAAGCATGTTGCA | GCAATAGGTCATGCATATATCACAGT |
|  |  |  | H | GAGAAACAGACTTTCATGTGCTCC |  |  |
| PgFD118 | G | A | F | ATTTCCTGATGGATGCTTTGCG | TCCCAAGTCGTGGCTTACCA | GGCTTTAACAAAGGTTGGGCA |
|  |  |  | H | CATTTCCTGATGGATGCTTTGCA |  |  |
| PgFD119 | A | T | F | CGGGTGAGATAACGGTGGAAAT | CTCCATGCCTCTACTAAGAGTAGAGGT | CTCGCATTGTCCAATGTAAGATCTA |
|  |  |  | H | CGGGTGAGATAACGGTGGAAAA |  |  |
| PgFD120 | A | G | F | AGAGATTATGAGCATTTGAAGAATCCCA | TCTTGTATGCTAGCTGGAGGCATT | TGCCTTTCAGTGTCATCAAGAAAT |
|  |  |  | H | GAGATTATGAGCATTTGAAGAATCCCG |  |  |
| PgFD121 | T | G | F | ATCCTTCGAAATGCATCAGACAGTA | ACCTTTCGACGAAGGTAAAAGTTCTACG | CATGGATCGCAGAGTAAGCAT |
|  |  |  | H | CCTTCGAAATGCATCAGACAGTC |  |  |
| PgFD123 | C | A | F | GACGGATATTCCGTCGATTTTATGC | CACTTATCAAATTTATGCTCGTGTGGGA | CGACGGACCAACCTTTATTTCC |
|  |  |  | H | CGACGGATATTCCGTCGATTTTATGA |  |  |
| PgFD124 | A | C | F | GTTGGGTGTAATCTAACCTGCAATAT | CCAGAATTTCCTAAATACAAGATTCGGCAT | TGAGCATGCATTAGTTTTTCTCCA |
|  |  |  | H | GTTGGGTGTAATCTAACCTGCAATAG |  |  |
| PgFD125 | A | G | F | GTGATTGTATAGGTCCATCGATTCATTTT | ACACCTCTAACTACAAATGATAAAATATACTAGTATTAAA | TGCATCAAAAAGATGTTTGCAAGTG |
|  |  |  | H | GTGATTGTATAGGTCCATCGATTCATTTC |  |  |
| PgFD127 | C | T | F | GGATAATTTTCCTGCTCGGTCG | GTTCGGGAAGCCCAAGCAA | AGGATGAAGTTCTCGAATATTTCATGG |
|  |  |  | H | GGGATAATTTTCCTGCTCGGTCA |  |  |
| PgFD128 | A | G | F | AAGCTGTACTAAGCCTTGCCATA | ATGACAAGCTTCCCTCAGACAACT | GGGAATGATATTGGCACAGATGA |
|  |  |  | H | GCTGTACTAAGCCTTGCCATG |  |  |
| PgFD129 | C | T | F | GCAGATAAGATATACCATCCGACAACTAC | CGTTTCTTAAAATAACATATATTAAGGAACGGAGGG | GCCGATGATGAATCATATCCGTAT |
|  |  |  | H | GCAGATAAGATATACCATCCGACAACTAT |  |  |
| PgFD130 | C | T | F | AAATATTACAACAATAATCATGCACCCATAAAATTC | TTATAATCAAAGTTTTGAATTTGGTTTGATTTGTGTGATT | GTAATTTGGAAGTTCTTACATGATCAACA |
|  |  |  | H | AAATATTACAACAATAATCATGCACCCATAAAATTT |  |  |
| PgFD131 | C | T | F | GCTGCTGCCAGAATTCTCTC | CCCCTCATGGGTTGCCCATA | CGGATGGTTCCTTCTCAGTCA |
|  |  |  | H | GGCTGCTGCCAGAATTCTCTT |  |  |
| PgFD132 | C | G | F | ACTTCGAGGTCAACTTCTTCTGAC | TGGTGACTTATAATGTAGCCAACACCT | CAAGAATTCCTGCTAACAAGCCT |
|  |  |  | H | CTACTTCGAGGTCAACTTCTTCTGAG |  |  |
| PgFD133 | T | C | F | CAAGAAGGATGGCAAAATTTACTTGGA | CTCCCTCAGATTCCCAGTGCT | GCAGTCCTAGTTACAAGTTATAGGAGA |
|  |  |  | H | AAGAAGGATGGCAAAATTTACTTGGG |  |  |
| PgFD134 | G | T | F | CCCTGGTGAATTCGTCATTGTC | CGAGTTCAGAAGGCGCTGG | GTAAATTCGCAAGGCAAATGAATCC |
|  |  |  | H | GCCCTGGTGAATTCGTCATTGTA |  |  |
| PgFD135 | T | C | F | GATTCTCCAAGCTACGGCTACTA | GCTCGAACACGTGTCGCT | TTCCTTCTTCTTCCTTTCTCCTGA |
|  |  |  | H | ATTCTCCAAGCTACGGCTACTG |  |  |
| PgFD136 | A | G | F | GCCACCAAATGGCCCGAT | ACTTGACAAACGGGTCGGGT | TCGCTCCATCAAAATTTGGACC |
|  |  |  | H | CCACCAAATGGCCCGAC |  |  |
| PgFD137 | C | T | F | CAAGGTTTTTGTTGTTCAACCCG | TGAACAAAAAGATGTTCAAATAAAGAAAGTAGAGCAA | GCTCTCATGTCTCGTGTAATTCC |
|  |  |  | H | CCAAGGTTTTTGTTGTTCAACCCA |  |  |
| PgFD138 | C | T | F | GTCGGACAAAAAATGACCCGG | CGAGCCACGGGCCTCA | TGTAGACCTGGCAAACGAGT |
|  |  |  | H | GTCGGACAAAAAATGACCCGA |  |  |
| PgFD140 | G | A | F | GGTGTCATCTTAGTTTCAAGCCG | CCAGCTCAACCTAGCCCGA | ACTTAGGTGGGTTGGGCA |
|  |  |  | H | AGGTGTCATCTTAGTTTCAAGCCA |  |  |
| PgFD141 | C | T | F | TGTGGCACCGTCCGAG | CCCTTTCCGGCATCATCCC | ACACATTGTTGTACGTCGAAAAGAT |
|  |  |  | H | TTGTGGCACCGTCCGAA |  |  |
| PgFD142 | A | G | F | TTTCTGCTAAGAATTATTACAGGTCCCA | ACACGATAACTCCAATAATCCAACTGTTGA | ACATGAATGAAAGAAACAATGTTTTAAGAACT |
|  |  |  | H | TCTGCTAAGAATTATTACAGGTCCCG |  |  |
| PgFD144 | T | G | F | AAGAGAAATATGACCAACAGTAGTTCGAATATAT | GGGCAATATCTAATAGTAAGAAGTATAAAAAAAGAAATTC | CCCTGTATAGCTTCTTCGATTTCTC |
|  |  |  | H | AGAGAAATATGACCAACAGTAGTTCGAATATAG |  |  |
| pgFD145 | G | T | F | AACGTTGTCACTAAAATAGAACGAAATCG | GAGGAAATGCTTAGTTTAACTTAATATGTAATATATATGT | GGAAAGGCTGTTGTCACTGA |
|  |  |  | H | GAACGTTGTCACTAAAATAGAACGAAATCT |  |  |
| pgFD147 | G | T | F | GAAAAGATAAAACCAAAAAGGGATTCTTAATCC | ACCAAAAGAGGTTAATGACATGAGTTTTAAACTG | GCTTTATTCTTCTGAAGGTGGGA |
|  |  |  | H | AGAAAAGATAAAACCAAAAAGGGATTCTTAATCA |  |  |
| pgFD149 | T | A | F | ATCTCCCGGCCAACGTAATTTA | ACCCATATGAGATACAGAGGAATAGGCT | CTATGCAGCTTCAACATCTCCC |
|  |  |  | H | TCTCCCGGCCAACGTAATTTT |  |  |
| pgFD151 | G | T | F | ATGCTAATGGGCACATGTTGC | CCGCCAGCACAAAACTTGGA | CGGGTCAGTACTTTAGCATACATGA |
|  |  |  | H | GATGCTAATGGGCACATGTTGA |  |  |
| pgFD152 | T | G | F | CTTTTTAGCCGGGTATGGGTTAGATA | TCCCTACTACAAGCATCGCCG | GGCTGGTGTATTTTTTGGTTTCAT |
|  |  |  | H | TTTTAGCCGGGTATGGGTTAGATC |  |  |
| pgFD153 | A | G | F | AGGATTGTTTGTAGTCTTTGCAGATTTT | GGGTCCTCTTCTGACCGAAGAA | TTCGACCGAGAGGTCCG |
|  |  |  | H | AGGATTGTTTGTAGTCTTTGCAGATTTC |  |  |
| pgFD154 | G | A | F | CGAAGAAGGGGCAAAACAGTTC | CTCCCTGACTATCGGGCACT | CCAAGACTCAAATTCAGGCCTT |
|  |  |  | H | CGAAGAAGGGGCAAAACAGTTT |  |  |
| pgFD155 | C | G | F | ACTGAGCCCTTGGCCTAAAC | GGCGAGCACGAGTACAAAACA | CATCCACGTGTGGTTCTATCTAAAA |
|  |  |  | H | ACTGAGCCCTTGGCCTAAAG |  |  |
| pgFD157 | G | A | F | TGGGAATTCGTCGGGGC | CTGGTACTGAACTCGCCCTAGT | GGAAAAACCAAATGACCTTGGGA |
|  |  |  | H | CTTGGGAATTCGTCGGGGT |  |  |
| pgFD158 | C | T | F | CGTCTAGGAGTTCTACGCAAATACAG | GCCTCCAGACCGAATGGAACT | CGGATTTAGCAGTTGTGACAGT |
|  |  |  | H | CGTCTAGGAGTTCTACGCAAATACAA |  |  |
| pgFD159 | G | A | F | AGCATTCTGGGCTCGCC | GTTCTGCCCCAAGGGCAA | CAAAGAGTTAGGCATCGGGAA |
|  |  |  | H | AAGCATTCTGGGCTCGCT |  |  |
| pgFD160 | G | A | F | CTCCACAGCCATACCCAAAAC | CCATAAGGAGGGCGAGCGA | CAACCAAGACCTTCACCCC |
|  |  |  | H | CCTCCACAGCCATACCCAAAAT |  |  |
| pgFD161 | T | C | F | CTCTCCGGAAAGAGCACTAACATA | TCCTTCGAAAATAACGTAACTGACAAAAACTTC | CGGTCAAAATTATACTAACCTCTCCG |
|  |  |  | H | TCTCCGGAAAGAGCACTAACATG |  |  |
| pgFD162 | A | G | F | CAAGACCAACTTTAGGTTCGACTCT | ACGACCCGATCTTGGGGAC | GACACTCATCTTCTCCCGAATTG |
|  |  |  | H | AGACCAACTTTAGGTTCGACTCC |  |  |
| pgFD163 | T | C | F | CTCTTACAAGATCCTTACATAGAAATCCTCA | AGGGCAAAACTCAGAATCTGAATTCTCT | GTGTTGTCAACCCACAACAAAAC |
|  |  |  | H | TCTTACAAGATCCTTACATAGAAATCCTCG |  |  |
| pgFD165 | C | T | F | GGCGTTGGGATTCCTTTGTTAC | CACATTAGGCCAGCGCCG | CAACACTGCCTCCCAGC |
|  |  |  | H | GGCGTTGGGATTCCTTTGTTAT |  |  |
| pgFD166 | T | A | F | TTTTGGGATTCATTTAGCAACGAACAT | GACAGTTGTGTCCGCGCT | GTCGTCGCTAATTACAAGCTCAAT |
|  |  |  | H | TTTGGGATTCATTTAGCAACGAACAA |  |  |
| pgFD167 | G | T | F | GGTAGAGTTATAGGAATTCCATTGGTCC | CCAAGCTCTCGCAAAAGGGA | CTACAGGTGGACCAAAGAAGG |
|  |  |  | H | GGTAGAGTTATAGGAATTCCATTGGTCA |  |  |
| pgFD168 | T | A | F | TGAAGAAATTGGTCATCAACCTGAGAT | TGGACCATTTCAGTGCTCTCCT | ACACTTCATGTTCCCATTCGTG |
|  |  |  | H | TGAAGAAATTGGTCATCAACCTGAGAA |  |  |
| pgFD169 | A | C | F | GTCTGCCCCAGTGATTCCAT | CGCCAAGGCAAATTCAGCCA | TCATCTCTAATCAAGTCTGCCCC |
|  |  |  | H | TCTGCCCCAGTGATTCCAG |  |  |
| pgFD170 | C | T | F | TCCAATGTCTGTTCGAATCATACCAG | CCTACAATGAACTATTAGATGAGGACGATTTGA | AATTCCCAATCCGCAACCAT |
|  |  |  | H | TCCAATGTCTGTTCGAATCATACCAA |  |  |
| pgFD171 | G | T | F | AGAATTCTCAATGAGAGGTGCCTTC | GGGACACACACCTGAAGGTTC | AGCGTCTATCCTTCTCACCG |
|  |  |  | H | AAGAATTCTCAATGAGAGGTGCCTTA |  |  |
| pgFD172 | T | C | F | CAGGAGCCCTCCCTTGT | GTGGAAGCCGACACATCCAA | CCCCAAGAAGCATGGAGAATT |
|  |  |  | H | CAGGAGCCCTCCCTTGC |  |  |
| pgFD173 | A | G | F | GTAGTTGCTTTGTCGCCTGATTTA | GGCAGTGCACCCCCAA | CATGGAAGTGGTATTGAATTCAATGAT |
|  |  |  | H | AGTTGCTTTGTCGCCTGATTTG |  |  |
| pgFD174 | G | A | F | CTTCGACACAACCCTAAGGGAG | CCCCTAGGGTTAGCCCCTT | CATCGTCGAAAAGCTTCAGAGG |
|  |  |  | H | CTTCGACACAACCCTAAGGGAA |  |  |
| pgFD176 | C | G | F | CCTTCGAGAATTCAAATCCGAAGAG | CATTTTGCTCTGCCAATCTCCCA | CAACGGGAAATTTTTTGAAAGACCA |
|  |  |  | H | CCTTCGAGAATTCAAATCCGAAGAC |  |  |
| pgFD177 | G | A | F | AACACGTCTCGGATGTCGG | AATCATCCATCGCTCAGTCCCA | ACCCGAAGCTTGTACTCCTAAG |
|  |  |  | H | GAACACGTCTCGGATGTCGA |  |  |
| pgFD178 | T | C | F | CCACCACACGCCGAGTT | GTCTTTTCCAGTTTGGTACGTGCT | GGTCGTTTGAACCGCCTAT |
|  |  |  | H | CCACCACACGCCGAGTC |  |  |
| pgFD179 | G | C | F | GCCCAATTTATAATGTTAGGCCTATACG | ACGGGAATTCAACCGCGGA | GGTGCATGCTTGTTTTCGG |
|  |  |  | H | GCCCAATTTATAATGTTAGGCCTATACC |  |  |
| pgFD180 | A | G | F | CTCATCATCAGAGGAATCCAGGAT | ACAAGCTTCCGGCCTCGA | GTAAGAGTTACGCCTTAAGCTTCG |
|  |  |  | H | CTCATCATCAGAGGAATCCAGGAC |  |  |
| pgFD181 | G | A | F | GTGGGCCAAAAAAGCCCAG | ACGGGCCAGGTTTTGAGCTA | ACGGGTCGGACCAAAAATG |
|  |  |  | H | GTGGGCCAAAAAAGCCCAA |  |  |
| pgFD182 | A | G | F | CCCAGATCAGATTAGACAACCCA | CGTCGTTGTTGTCACTGCCA | TGATCGTCATCATCAGCCGA |
|  |  |  | H | CCCAGATCAGATTAGACAACCCG |  |  |
| pgFD183 | G | A | F | TCTAAGCAAATTATGGGGCTCTCTC | GCTTCCTAGACTTTGTAGAAGACAACTGC | AACTCTTACCAAGTCCCAAAATTGT |
|  |  |  | H | TCTAAGCAAATTATGGGGCTCTCTT |  |  |
| pgFD184 | G | A | F | CTCTCTAGTATACTCCATATCCTCCTCC | GGGGTATATATGTATTTGGATTTAGGGCTTTGA | TCCCTTATGTAGAATTCTCCTAATCCA |
|  |  |  | H | ACTCTCTAGTATACTCCATATCCTCCTCT |  |  |
| pgFD185 | A | G | F | CCAAGTCGGATGTTTTGCTTTGATAAA | GAGCTCATTTAAGAATTCAACTCGATTCGT | GGGAACCAAGTCGGATGTT |
|  |  |  | H | CCAAGTCGGATGTTTTGCTTTGATAAG |  |  |
| pgFD186 | C | A | F | TGGAGCTCACTTTGGGTGG | AGTCATGCCCTCCTCGCTT | GAACAGGTGATTTGGTGATCGT |
|  |  |  | H | TGGAGCTCACTTTGGGTGT |  |  |
| pgFD187 | T | C | F | CGATGGCGTCGAATTCTTCAAA | AGGGTCTAACAAACCACCCCC | GGGACTACTCAGAATTTCTTCGAC |
|  |  |  | H | CGATGGCGTCGAATTCTTCAAG |  |  |
| pgFD188 | T | C | F | ATTCAATGAATGGTCACTACGATCTATGA | CGCGTTCACCGACGAACA | GTTCGGATTGACTCTTGTGTGA |
|  |  |  | H | TCAATGAATGGTCACTACGATCTATGG |  |  |
| pgFD189 | T | C | F | CCGGCGAATTCGAGGTCA | CCGATCAAATTCCGGCAATTGGTA | TGTCTCGCAATCTCAAATTCACC |
|  |  |  | H | CCGGCGAATTCGAGGTCG |  |  |
| pgFD190 | G | T | F | GAGGTGTATAGCCTATAAGCTTCACATAC | AAAATGGAAAAGTTATTTACATGCTTGAGACAAGAT | CGAGTACATTTCCATGTTGCACA |
|  |  |  | H | GAGGTGTATAGCCTATAAGCTTCACATAA |  |  |
| pgFD191 | A | G | F | TTGATTTTTGATAATTTCTCAGGGCAATCT | GGAAACATCAAAGCAGGCCCC | TCTTGAGGGTTGTCTCTGAGC |
|  |  |  | H | TGATTTTTGATAATTTCTCAGGGCAATCC |  |  |
| pgFD192 | T | C | F | CTCTGGAGGTTATGCGTAACCA | TCAGACTCGCTGCGCTGATT | CGCTGAAAAGTAGAGCCATAATCT |
|  |  |  | H | TCTGGAGGTTATGCGTAACCG |  |  |
| pgFD193 | G | A | F | GCAGCTTCAGTGTGCGC | TGTCCGAGCTCGCCCC | TTGCCATACGAATGCACCG |
|  |  |  | H | GCAGCTTCAGTGTGCGT |  |  |
| pgFD194 | C | T | F | CGCGAACTTCTGTATGGAATTCG | CGGACCAAGAGGCTACCCA | CTCATTTGCTGTGGCGCT |
|  |  |  | H | CGCGAACTTCTGTATGGAATTCA |  |  |
| pgFD195 | G | A | F | GGGCCAGATCCCTCTTTTGG | AAGGGACTCGAATGCCCGA | TGGGAATCCGAGTCCTCTG |
|  |  |  | H | GGGCCAGATCCCTCTTTTGA |  |  |
| pgFD196 | C | T | F | ACTATAGCCGATGGTGCAGC | GGTTTGTTGCAACACCTGCG | GTCTCCCTGGAGTTAGCATAGAAT |
|  |  |  | H | CACTATAGCCGATGGTGCAGT |  |  |
| pgFD197 | G | A | F | GAGATAGACAACAGACCCGCC | CTTGCGGTGATTCTCAGCACAT | GATTCCTCTATAGTTCGTGGTGTTG |
|  |  |  | H | GAGATAGACAACAGACCCGCT |  |  |
| pgFD198 | G | A | F | CGGTCTTCATGAGCAAGGATGG | CCACTGCAGGGTCCGGT | GGTTGAGGCCTGATTCATTTGA |
|  |  |  | H | CGGTCTTCATGAGCAAGGATGA |  |  |
| pgFD199 | C | T | F | CCTCCCTTGAATTCATGTATTTCTTGC | ACCAAGGAAGTAGGGGCACAA | GGACCGACACAAAAGAAACATCC |
|  |  |  | H | CCTCCCTTGAATTCATGTATTTCTTGT |  |  |
| pgFD200 | G | A | F | GACAGACAGTCCAGGATACATCATC | CCTGCTCTCGGTCTGCCTA | AGGTGGAGGAATTCGCCA |
|  |  |  | H | GACAGACAGTCCAGGATACATCATT |  |  |
| pgFD201 | G | A | F | CTCCACCCGTGGCTAGAG | GGAGGAAATTACCAGGTAGATGCTTCA | GAATTCCTCCACCTTCGGC |
|  |  |  | H | CTCCACCCGTGGCTAGAA |  |  |
| pgFD202 | G | T | F | GTAATAAAGTGACCCAAGCCAATCG | AGCCTGACGAACTCGTGTTGA | CTCCACGTCGATACCCGT |
|  |  |  | H | GTAATAAAGTGACCCAAGCCAATCT |  |  |
| pgFD203 | T | C | F | AAAACGATCTTATCAGGTGGAGCT | TTCTTTACCATCCATCTCTTCCAACCTT | TTGGGAATGACCCTTTGTTGTC |
|  |  |  | H | AACGATCTTATCAGGTGGAGCC |  |  |
| pgFD204 | A | G | F | GTTGCTTTTATTGAAATCCCGGTGA | CCAAGCTCAACCACAAGTTCCA | TCGTGCTGAGGAAGTTGCTA |
|  |  |  | H | TTGCTTTTATTGAAATCCCGGTGG |  |  |
| pgFD205 | A | G | F | CGAGTCTACCACAAGTAGACCCA | GGGTCTACCCGTAGCAGCC | CCACGGGACTGAAACCAC |
|  |  |  | H | GAGTCTACCACAAGTAGACCCG |  |  |
| pgFD206 | T | C | F | TCCAAGTCCTACTACCGGCA | TGGCACAACTGCGCCATTT | CATGCTCTTAGTCGAAATTATCTTCCA |
|  |  |  | H | CCAAGTCCTACTACCGGCG |  |  |
| pgFD207 | A | G | F | AGGCTTGTGAATTCGCCGA | ACTTGTCCCAACCCATTCCTCT | CGACGAATTCGCCGGG |
|  |  |  | H | GGCTTGTGAATTCGCCGG |  |  |
| pgFD208 | G | A | F | ACAAGTTTTCCAATGCACAGCAC | GGTGCATGTCGGTTAGCCTT | GTGCATATTCCAACTATTCCCAGAT |
|  |  |  | H | ACAAGTTTTCCAATGCACAGCAT |  |  |
| pgFD209 | G | A | F | TGGCCACAATTCGGGACAG | CGCCCTCGTCGGGTGAT | GGCAGTGGACTACACTTGAG |
|  |  |  | H | TGGCCACAATTCGGGACAA |  |  |
| pgFD211 | G | A | F | ACCCATGTATGAAAAGTCTTACTCGATC | GCCAACTATGTCAAGGCAGACAC | GGGATGATTTGTGTCAGACCC |
|  |  |  | H | ACCCATGTATGAAAAGTCTTACTCGATT |  |  |
| pgFD213 | C | T | F | TGTTCCCTTTGTCCCCCC | TGACACATAATTTTTTATTTAATACCAATTAACTCTAAAA | TCCTCTATCCCCAATTTGCCT |
|  |  |  | H | CTGTTCCCTTTGTCCCCCT |  |  |
| pgFD214 | A | G | F | TCCTTTGACCTTCGTGGACAA | CCAATGCATCGATAGATTTTGGGCT | TGATGTCTGATTCTTTCATTTTCAGACT |
|  |  |  | H | TCCTTTGACCTTCGTGGACAG |  |  |
| pgFD215 | T | C | F | CGGAATTCCATGCAATCTTACCCTA | GCTATACGTGGGCGCGG | GTGAAAATTTGGCGCGCTT |
|  |  |  | H | GGAATTCCATGCAATCTTACCCTG |  |  |
| pgFD216 | C | T | F | GTGTATGGAATTCGGTTACTGCG | CCAAGTCGAGTCGTAGTGCAGA | GCGGCGCGAGTTAATAGT |
|  |  |  | H | AGTGTATGGAATTCGGTTACTGCA |  |  |

^a^ FAM; Fluorescein amidite

^b^ HEX; Hexachloro-fluorescein

**Table S8.** Summary of ginseng germplasms applicated to SNP chip.

| **Type** | **Country** | **Number of germplasms** | **Remarks** |
| --- | --- | --- | --- |
| Cultivar | Korea | 15 | Registered in Korea Seed & Variety Service |
|  | Japan | 3 | Collected from Japan |
|  | China | 15 | Collected from various regions in China |
| Breeding line | Korea | 589 | Bred and maintained in Rural Development Administration, Korea |
|  | China | 4 | Bred and maintained in Rural Development Administration, Korea |
| Wild-simulated ginseng | Korea | 171 | Collected from various regions in Korea |
|  | China | 49 | Collected from various regions in China |
|  | Russia | 9 | Collected from Vladivostok, Russia |
| Wild ginseng | Korea | 43 | Collected from various regions in Korea |
|  | China | 21 | Collected from various regions in China |
| Total |  | 919 |  |

**Table S10.** The number of ginseng genotypes included in each group per classification.

| **Classification** | **Group1** | **Group2** | **Group3** | **Group4** |
| --- | --- | --- | --- | --- |
| **Cultivar** | 1 | 2 | 9 | 41 |
| **Breeding line** | 41 | 58 | 164 | 330 |
| **Wild-simulated ginseng** | 11 | 11 | 51 | 131 |
| **Wild ginseng** | 4 | 2 | 17 | 46 |
| **Total** | 57 | 73 | 241 | 548 |
|  | 919 | | | |

**Figure legends**

**Fig. S1.** Diagram illustrating the linkage of markers between genetic map and physical map of *P. ginseng*. The genetic map is labeled with LG, and the physical map is labeled with chromosome IDs of the reference telomere-to-telomere genome. Each linkage group on the genetic map corresponds to a specific chromosome on the physical map.

**Fig. S2.** Scheme of GBS data handling pipeline for SNP discovery (A) and SNP chip development using high-fidelity sub-genome unique SNPs based on GBS data (B).

**Fig. S3.** Endpoint fluorescence scatter plot of the KASP markers applied to ginseng germplasms. The X and Y axes of endpoint fluorescence scatter plot represent the FAM (465-510 nm) and HEX (533-580 nm) values, respectively, which represent the genotype of the SNPs.

**Fig. S4.** Two sets of SNP chip performance report (A) and gathered scatter plot of each marker set (B). Each set of SNP chip comprises 96 SNPs selected from a pool of 192 final SNPs.

**Fig. S5.** Delta K graph draw based on results of population structure analysis with total 919 germplasms.

**Fig. S6.** Heatmap of Pearson correlation coefficients between 192 marker genotypes and phenotypic traits across 119 germplasms.

**Fig. S7.** Cross-species SNP chip application. (A, B) Population genetic analysis of 919 ginseng and 19 P. quinquefolius with UPGMA phylogenetic tree and population structure plot. (C) Number of null genotypes among 192 SNPs of *P. ginseng*, *P. quinquefolius* and *P. vietnamensis*. (D) Phylogenetic tree based on chloroplast genome. Reorganized and redrawn based on divergence time estimations of Kim et al. (2018) [4].

**Fig. S1**


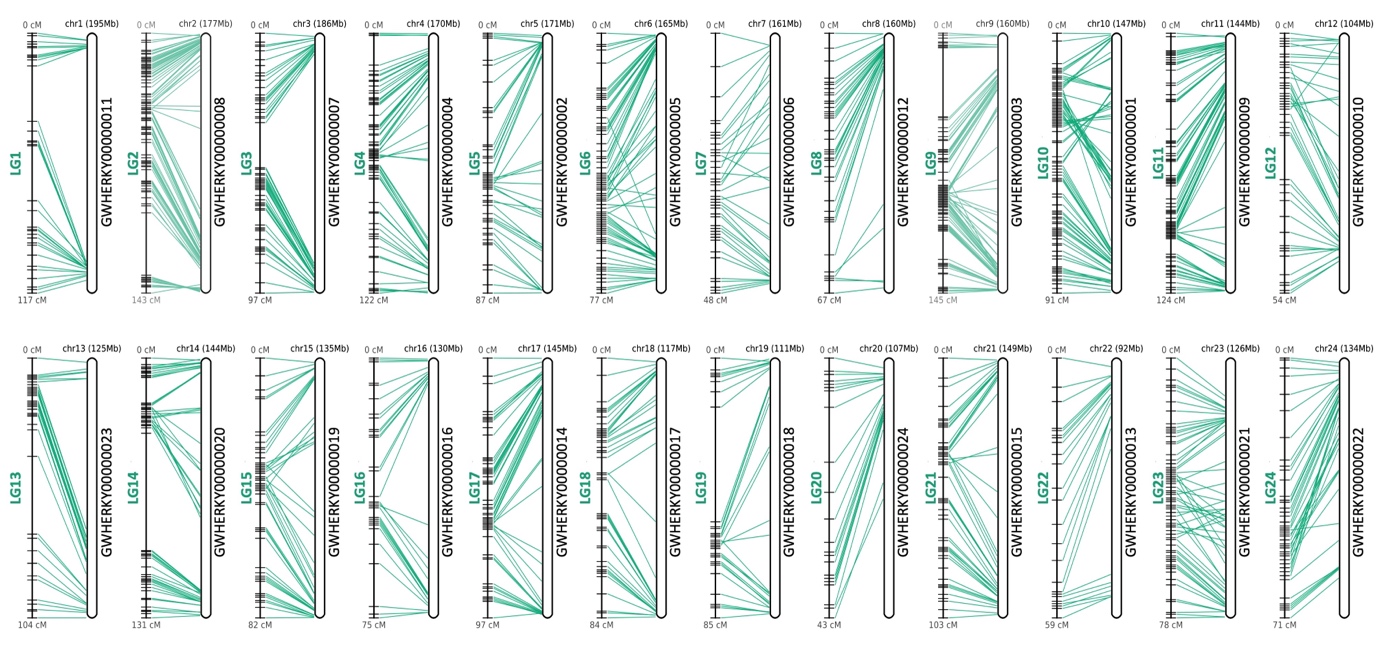


**Fig. S2**


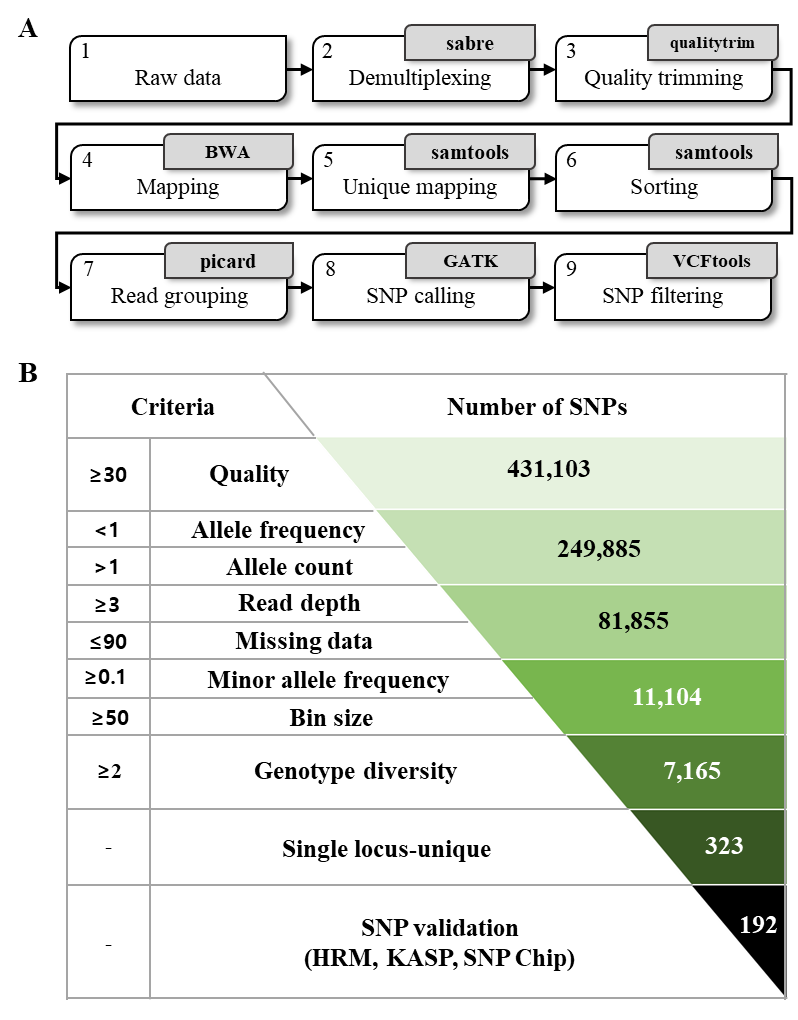


**Fig. S3**

**
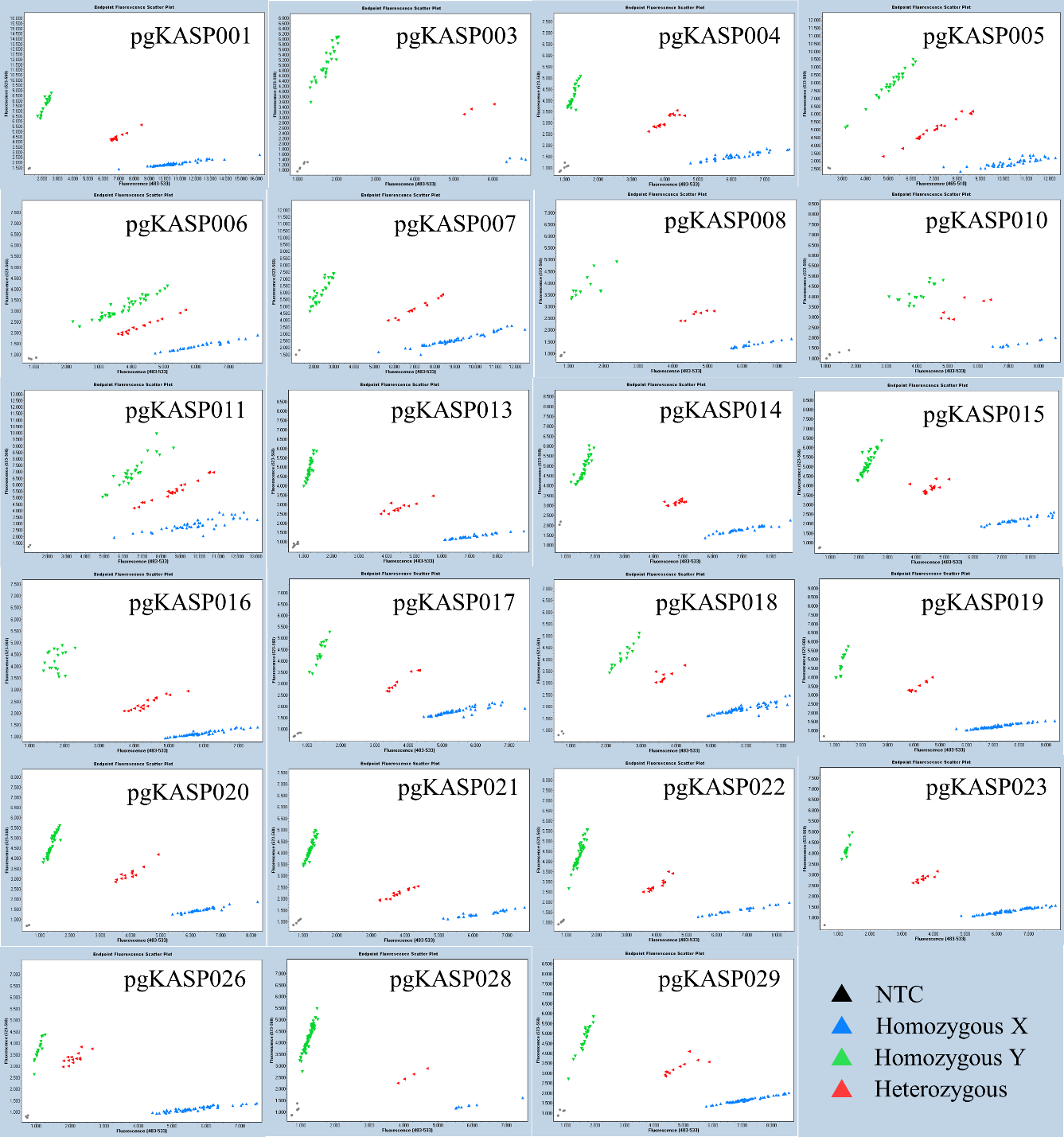
**

**Fig. S4**


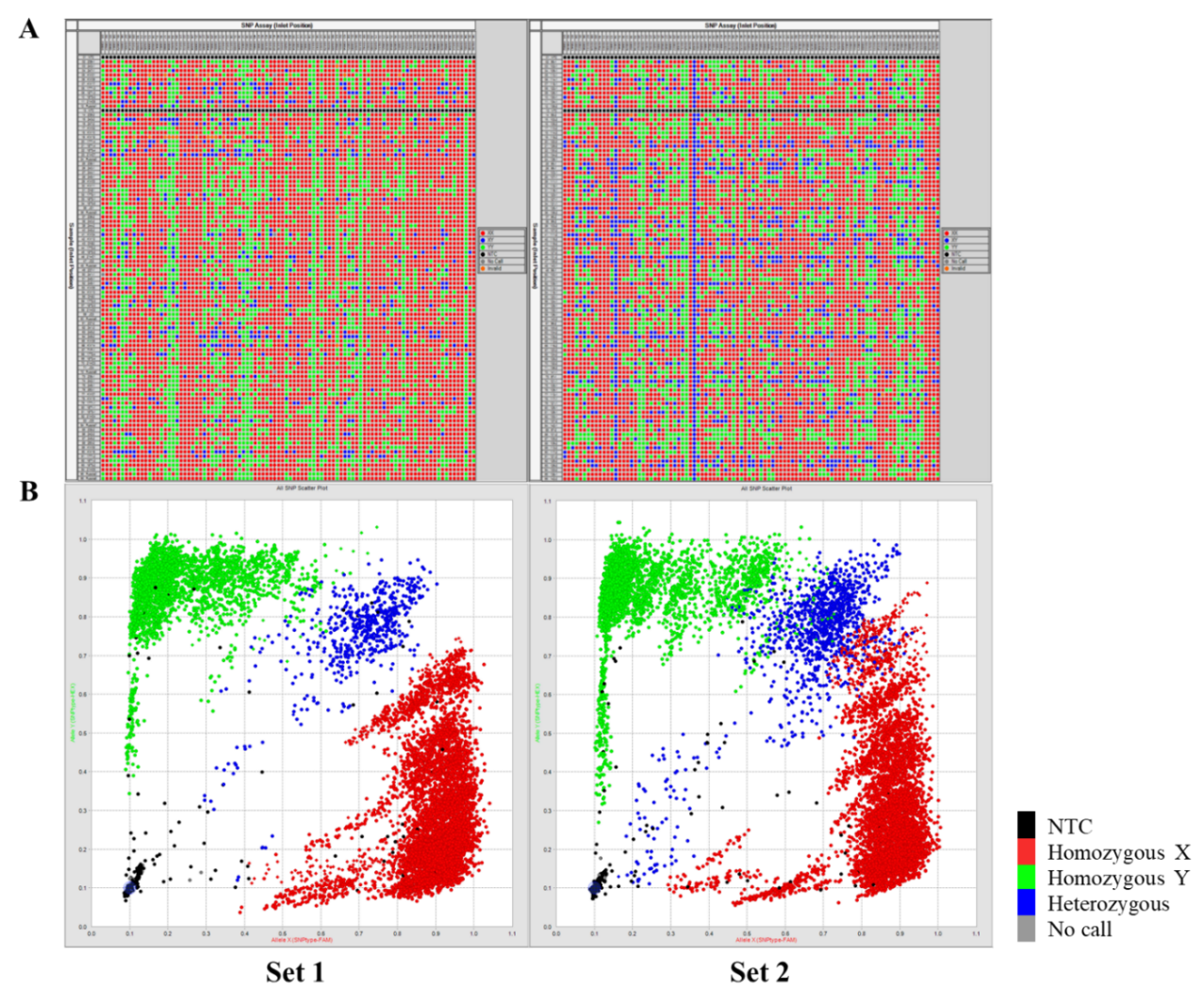


**Fig. S5**


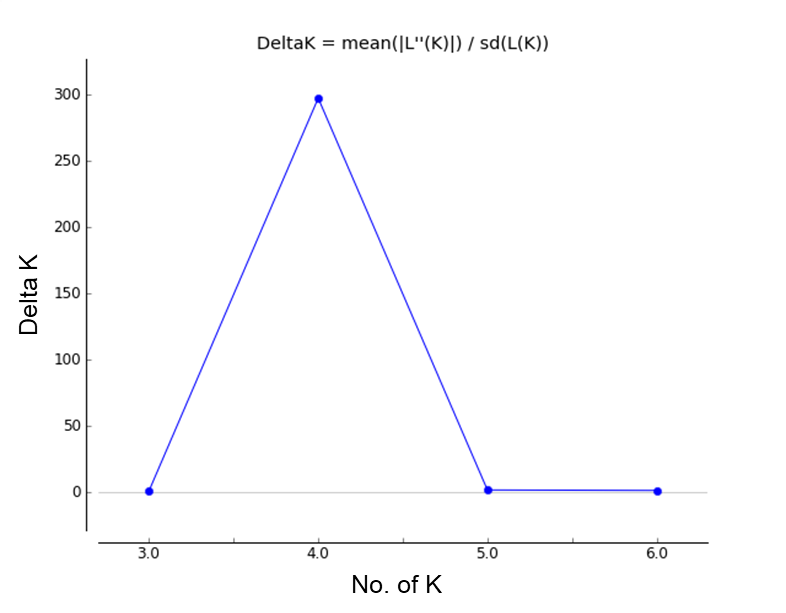


**Fig. S6**

**Fig. S7**


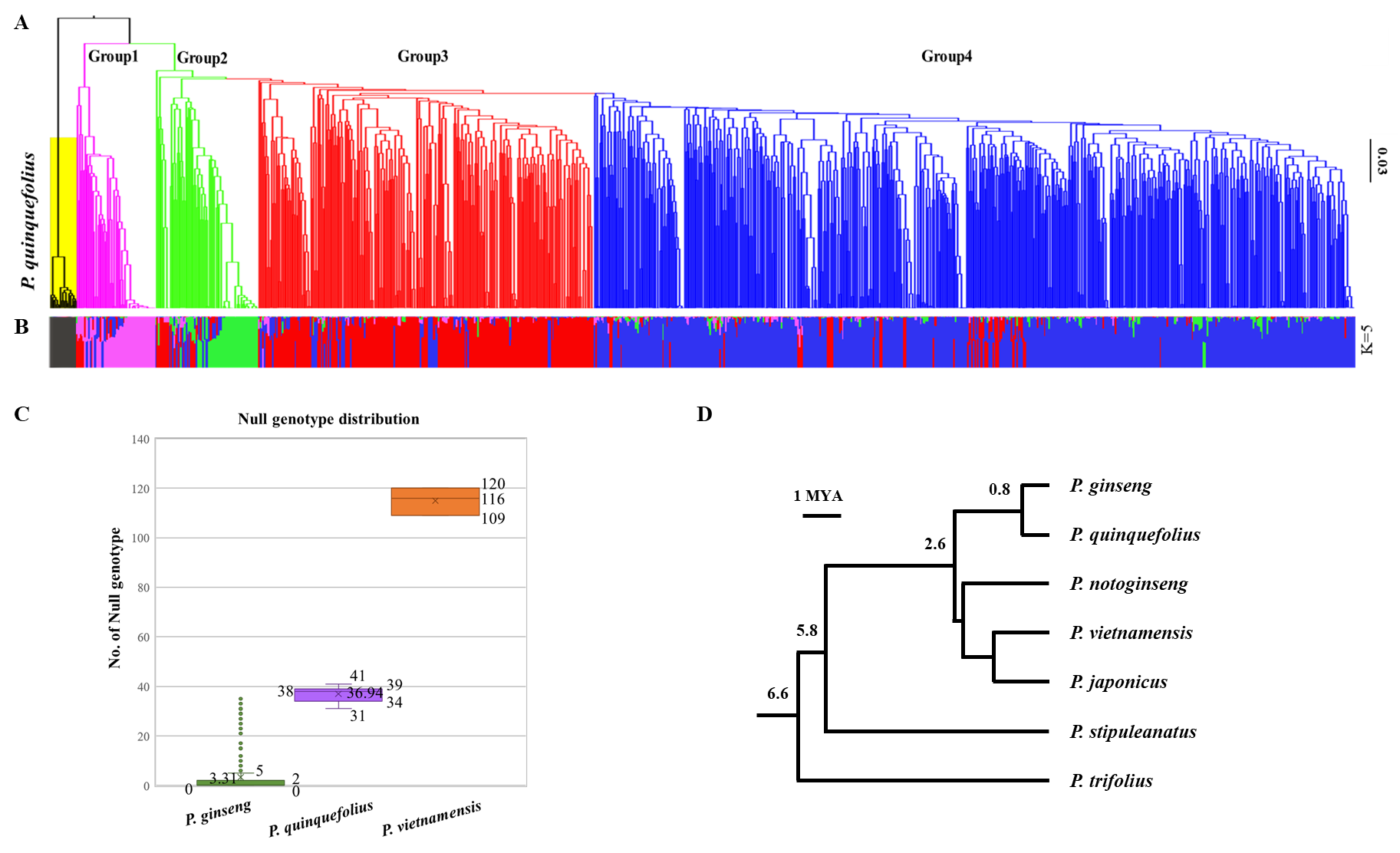

Supplement: Web_Material_uhae257 [file web_material_uhae257.zip › SNPchip_Supplementary_Table_Figure_2nd_revision.docx]
